# Changes in the structure and function of rhizosphere soil microbial communities induced by *Amaranthus palmeri* invasion

**DOI:** 10.3389/fmicb.2023.1114388

**Published:** 2023-03-28

**Authors:** Mei Zhang, Cong Shi, Xueying Li, Kefan Wang, Zhenlu Qiu, Fuchen Shi

**Affiliations:** ^1^Department of Plant Biology and Ecology, College of Life Sciences, Nankai University, Tianjin, China; ^2^School of Environmental Science and Engineering, Tiangong University, Tianjin, China

**Keywords:** plant invasion, *Amaranthus palmeri*, keystone taxa, functional genes, molecular ecological network

## Abstract

**Introduction:**

Plant invasion can profoundly alter ecosystem processes driven by microorganisms. The fundamental mechanisms linking microbial communities, functional genes, and edaphic characteristics in invaded ecosystems are, nevertheless, poorly understood.

**Methods:**

Here, soil microbial communities and functions were determined across 22 *Amaranthus palmeri (A. palmeri)* invaded patches by pairwise 22 native patches located in the Jing-Jin-Ji region of China using high-throughput amplicon sequencing and quantitative microbial element cycling technologies.

**Results:**

As a result, the composition and structure of rhizosphere soil bacterial communities differed significantly between invasive and native plants according to principal coordinate analysis. *A. palmeri* soils exhibited higher abundance of Bacteroidetes and Nitrospirae, and lower abundance of Actinobacteria than native soils. Additionally, compared to native rhizosphere soils, *A. palmeri* harbored a much more complex functional gene network with higher edge numbers, average degree, and average clustering coefficient, as well as lower network distance and diameter. Furthermore, the five keystone taxa identified in *A. palmeri* rhizosphere soils belonged to the orders of Longimicrobiales, Kineosporiales, Armatimonadales, Rhizobiales and Myxococcales, whereas Sphingomonadales and Gemmatimonadales predominated in the native rhizosphere soils. Moreover, random forest model revealed that keystone taxa were more important indicators of soil functional attributes than edaphic variables in both *A. palmeri* and native rhizosphere soils. For edaphic variables, only ammonium nitrogen was a significant predictor of soil functional potentials in *A. palmeri* invaded ecosystems. We also found keystone taxa in *A. palmeri* rhizosphere soils had strong and positive correlations with functional genes compared to native soils.

**Discussion:**

Our study highlighted the importance of keystone taxa as a driver of soil functioning in invaded ecosystem.

## 1. Introduction

Invasion of exotic plants is thought to be a result of globalization, agriculture and human activity (Seebens et al., 2015; Linders et al., 2019; Jin et al., 2022). The loss of native biodiversity caused by plant invasion has a significant negative impact on ecosystem processes and services (Craig et al., 2015; Wang et al., 2020; Zhang and Suseela, 2021). Microorganisms can either respond to plant invasion or cause it (Zhang G. L. et al., 2022). The soil microbiome is involved in numbers of biogeochemical processes in terrestrial ecosystems (Wang et al., 2019), including organic carbon (C) decomposition, carbon fixation, the nitrogen (N) cycle, the phosphorus (P) cycle, and the sulfur (S) cycle (Lynn et al., 2017; Xiang et al., 2020; Cheng et al., 2021). These processes are crucial in the survival and success of alien plants (Anthony et al., 2020; Yin et al., 2020). There is growing proof that alien plants modify microbial functioning in many ways, such as altering microbial assemblages to promote their successful invasion (Huang et al., 2016; Kamutando et al., 2019; Zhao et al., 2019; Sun et al., 2022). It is yet unknown, nevertheless, how these alterations take place under plant invasion settings and whether these modifications apply to all invasive species or only some of them (Nasto et al., 2022). Therefore, understanding the invasive mechanisms and evaluating their impact on environmental function may greatly benefit from revealing how the functional potentials of microbial communities respond to plant invaders (Shannon-Firestone et al., 2015; Rodríguez-Caballero et al., 2017).

In addition to forming ecological clusters and coexisting closely with one another, soil microorganisms also engage in a variety of partnerships, including competitive and cooperative ones (Berry and Widder, 2014; Ding et al., 2015a). The efficiency of resource transmission and ecological processes can both be improved by greater cooperation among microbial species (Lin et al., 2021). Recent research has used ecological co-occurrence networks and their topological features to examine the complexity and connectedness between taxa, taxa and edaphic qualities, or taxa and functional genes. These topological variables include modularity, net average degree, net path length, clustering coefficient and so on (Cordero and Datta, 2016; Shi et al., 2020; Xu et al., 2022). Co-occurrence patterns have been shown to be effective in examining the structure of microbial communities at various taxonomic levels, opening up fresh insights into potential interaction networks, and locating the keystone taxa in challenging situations (Ma et al., 2016; Fan et al., 2021). Understanding the composition and stability of microbial communities and their contributions to ecosystem processes depends on understanding the keystone taxa, which are highly connected taxa that, alone or in a guild, have a high explanatory power of network structure and function, regardless of their abundance (Berry and Widder, 2014; Chen et al., 2019). Numerous studies have shown that keystone OTUs may influence nutrient cycling and the functional stability of subterranean systems more than other OTUs due to their functional characteristics (Cheng et al., 2022). But we still do not know whether the introduction of foreign plants has an impact on the keystone taxa.

In the Jing-Jin-Ji regions of North China, *Amaranthus palmeri* (*A*. *palmeri*) encroaches into several habitats, posing a large threat and damage to agricultural security and ecosystem functions. *A*. *palmeri* adapts to environmental changes in heterogeneous habitats by controlling functional features, such as the leaf C/N ratio (Zhang et al., 2020). In the meantime, their invasion alters the enzyme stoichiometry and soil properties, providing favorable conditions for their own development and reproduction (Zhang M. et al., 2022). However, to our knowledge, no one has explored the underlying mechanisms linking keystone taxa, functional genes and edaphic properties in *A*. *palmeri*-invaded ecosystems. In the present study, we collected rhizosphere soil samples from 22 adjacent pairs of *A*. *palmeri* and native vegetation across a large spatial scale in North China. We used high-throughput sequencing and the QMEC approach to analyze the soil bacterial communities and functions involved in CNPS cycle. The aims of the present study were to (1) investigate the effects of *A*. *palmeri* invasion on the community compositions and microbial functions. (2) identify the keystone taxa and test whether the keystone taxa of *A*. *palmeri* would differ from native plants. (3) explore the major factors that influence microbial functional potentials in the invaded ecosystems. Our study may offer thorough understandings of the influencing elements and mechanisms of the soil microbes’ functional capacities during the invasion of *A*. *palmeri*, which may provide crucial details about possible management measures for *A*. *palmeri*.

## 2. Materials and methods

### 2.1. Sampling site and soil collection

This study was carried out at 22 sites (with each site including adjacent pairs of *A*. *palmeri* and native plants) located in Beijing-Tianjin-Hebei Province, China (38.74°–40.04°N, 116.36°–117.85°E) (Supplementary Table S1). Forty-four rhizosphere soil samples were taken from 22 sites (Supplementary Figure S1) in September 2021. All soil samples were delivered right away to the lab in an ice box. To get rid of stones and plant materials, soil samples were sieved (~2 mm). A portion of the soil was utilized to determine the soil’s physical and chemical qualities, while another portion was kept in a freezer at −20°C for DNA extraction. The soil pH, total carbon (TC), total nitrogen (TN), total phosphorus (TP), ammonium nitrogen (AN), nitrate nitrogen (NN) and available phosphorus (AP) content were determined. Our earlier investigations provided comprehensive descriptions of the study locations, sample collection, and measurement of physicochemical properties (Zhang M. et al., 2022).

### 2.2. DNA extraction, PCR, and high-throughput Illumina sequencing

Total genomic DNA was isolated from 0.5 g of soil samples according to the kit’s (MagaBio Soil Genomic DNA Purification Kit) instructions. NanoDrop One (Thermo Fisher Scientific, Waltham, United States) instrument was used to measure the total quantity and purity of DNA. Amplification of the V3-V4 region of the bacterial 16S rRNA gene using the primers 338F-806R (Cui et al., 2017) was performed using the Illumina MiSeq instrument (Illumina, San Diego, CA, United States) by Guangdong Magigene Biotechnology Co., Ltd. (Guangzhou, China). Primers were synthesized by Invitrogen (Invitrogen, Carlsbad, CA, United States). PCRs, containing 25 μl 2x Premix Taq (Takara Biotechnology, Dalian Co., Ltd., China), 1 μl each of the forwards and reverse primers (10 μM) and 50 ng of template DNA in a volume of 50 μl were amplified by thermocycling: 5 min at 94°C for initialization; 30 cycles of 30 s denaturation at 94°C, 30 s annealing at 52°C, and 30 s extension at 72°C, followed by 10 min final elongation at 72°C. The PCR instrument was a Bio-Rad S1000 (Bio-Rad Laboratory, CA, United States). Sequencing techniques and data analyses were also performed by Guangdong Magigene Biotechnology Co., Ltd. (Guangzhou, China). Trimmomatic software was used for the quality filtering of the raw fastq files. The pair-ended sequences were assembled using FLASH software after removing the primers and nucleotide barcodes. Subsequently, UPARSE was used to cluster these sequences into operational taxonomic units (OTUs) with a sequence similarity threshold of 97% (Edgar, 2013). Blast was used to annotate the representative sequences taxonomically (Geng et al., 2022).

### 2.3. Quantitative microbial element cycling

Quantitative microbial element cycling (QMEC) was further applied to illustrate the patterns of soil microbial functional-gene structures in the SmartChip Real-Time PCR System (WaferGen Biosystems United States) following the manual’s instructions (Zheng et al., 2018). QMEC, a high-throughput qPCR chip, contains 71 prokaryotic microbial CNPS genes, including 35\u00B0C-cycling genes, 22 N-cycling genes, 9 P-cycling genes, 5 S-cycling gene primers and one 16S rRNA gene primer (Supplementary Table S2). The amplification conditions were as follows: predenaturation at 95°C for 10 min was followed by 40 cycles of denaturation for 30 s at 95°C, annealing for 30 s at 58°C and extension for 30 s at 72°C. WaferGen software was used to perform qPCR and fluorescence signal detection, and automatically generated amplification and melting curves. When the amplification efficiency was less than 1.8 or greater than 2.2, the gene was eliminated. For quality assurance, the C_T_ values were collated, and a C_T_ of 31 served as the detection threshold. The absolute 16S copy number was utilized as a reference to determine the absolute abundances of functional genes (Zhu et al., 2017).

### 2.4. Statistical analysis

Taxonomic richness, Chao1, Ace, Shannon diversity, and Simpson diversity indices were calculated in the R 4.0.5 statistical environment (R Core Team, 2013)^1^ after rarefying the number of sequences per sample. The bacterial beta diversity was examined using principal coordinate analysis (PCoA) by calculating the Bray-Curtis distance matrices. The analysis of similarities (ANOSIM) method was used to assess significant differences in the bacterial community composition between invaded and native rhizosphere soils. The R package ggplot2 was used to display histogram plots of the soil bacterial community at the phylum (top 10) and family (top 20) levels across the 22 sites. Nonparametric statistical tests were applied to evaluate the community properties of invaded and native rhizosphere soils (e.g., significant differences at the phylum and family levels) (Kruskal-Wallis test, *p* < 0.05) in R 4.0.5. The Kruskal-Wallis test was also used to investigate the statistical significance of the relative abundance of functional genes in the north and south areas. The connections between the average standardized abundance of keystone taxa and the abundance of soil functional potentials were examined by linear regressions (OriginLab, United States). Both keystone OTUs and functional genes were ln transformed to make them close to normal distribution. The inverse distance weighted (IDW) approach was applied to examine the spatial distribution of soil functional genes using ArcGIS 10.2 (Environmental Systems Research Institute, United States). Random forest analysis was used to determine the contributions of soil physicochemical properties and keystone taxa on the soil microbial functional genes (Breiman, 2001). This analysis was performed using the lm and calc.relimp functions in the “relaimpo” package using R software Version 4.0.5.

Co-occurrence networks of soil functional genes were constructed with the “picante,” “reshape2” and “dplyr” packages in R 4.0.5 based on the Spearman correlation matrix. Functional molecular ecological networks between functional genes and OTUs were generated (fdr-adjusted *p*-values <0.05, correlation coefficients >0.6) (Zhu et al., 2017). OTUs or functional genes that were present in more than 80% of all samples were chosen to ensure reliable correlation. Gephi 0.9.2 was used to visualize each network (Bastian et al., 2009). The “multifunc” package was used to calculate the co-occurrence networks’ topological properties, such as degree, transitivity, and modularity. Keystone OTUs were identified as the top 5 degree centralities of OTU nodes (Xu et al., 2021).

## 3. Results

### 3.1. Bacterial abundance, diversity and community assemblage

**Figure 1:**
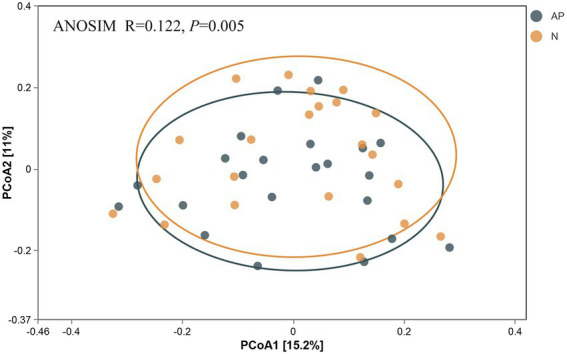
Bacterial community beta diversity. PCoA analysis of bacterial community in *A*. *palmeri* (AP) and native(N) rhizosphere soils, respectively; *R* and *p* values were calculated *via* ANOSIM test.

**Figure 2:**
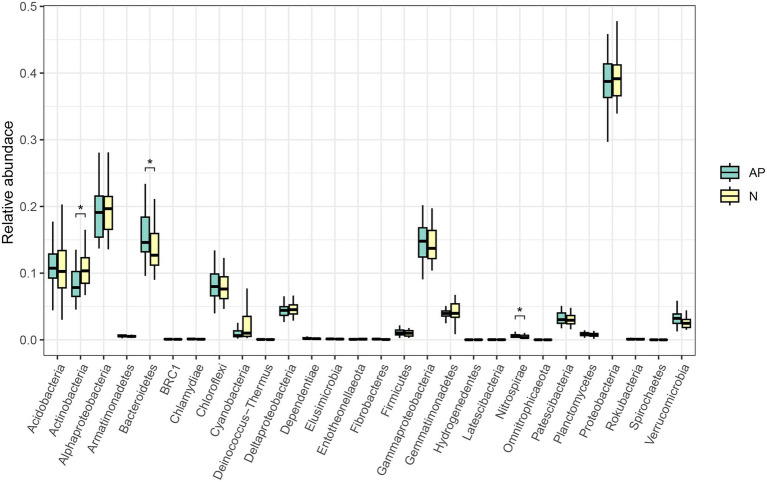
Kruskal-Wallis test at the phylum level of *A*. *palmeri* (AP) and native (N) rhizosphere soils. Significant level: * *p* < 0.05.

No significant difference was observed in alpha diversity (richness, Chao1, ACE, Shannon and Simpson indices) between *A*. *palmeri* and native rhizosphere soils (Supplementary Table S3). PCoA and ANOSIM analysis revealed a significant difference in β-diversity of soil bacterial communities between invasive and native rhizosphere soils (*R* = 0.122, *p* = 0.005, Figure 1). Proteobacteria, Bacteroidetes, Acidobacteria and Actinobacteria were the top 4 most abundant phyla in *A*. *palmeri* and native rhizosphere soils, accounting for 75% of the relative abundance (Supplementary Figure S2). The relative abundance of Bacteroidetes and Nitrospirae increased dramatically, while Actinobacteria declined significantly in *A*. *palmeri* rhizosphere soils compared with native rhizosphere soils (*p* < 0.05, Figure 2; Supplementary Table S4). The bacterial communities of *A*. *palmeri* and native rhizosphere soils at the family level consisted mainly of Sphingobacteriaceae, Chitinophagaceae and Burkholderiaceae (Supplementary Figure S3). Except for Nitrospiraceae, Kruskal-Wallis analysis revealed no significant difference in the relative abundance of the top 20 phyla between *A*. *palmeri* and native rhizosphere soils, while pronounced changes in less abundant families, including Archangiaceae, Azospirillaceae, Deinococcaceae, Fimbriimonadaceae, and Solirubrobacteraceae were observed (Supplementary Table S5).

### 3.2. Functional gene profiles of rhizosphere soil

**Figure 3:**
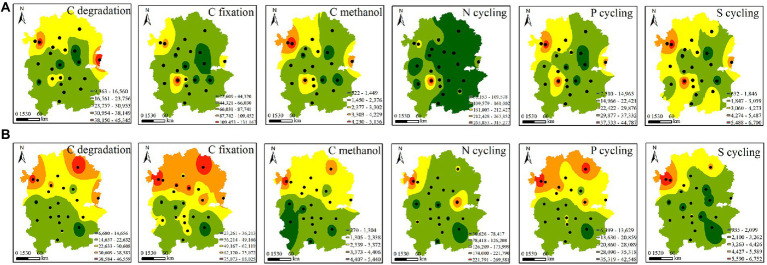
Spatial distribution of the abundances of the functional genes involved in CNPS cycles in *A*. *palmeri* **(A)** and native **(B)** rhizosphere soils. Black dots indicate the sampling sites.

Functional gene profiles were analysed by quantitative microbial element cycling (QMEC) technology. A total of 52 functional genes involved in N, C, P, and S cycling were measured in 44 soil samples. We identified 26 genes involved in C cycling, including C-degradation genes (12), C-fixation genes (10), and methane-metabolism genes (4). In addition, we also identified 16, 6 and 4 functional genes involved in N cycling, P cycling and S cycling, respectively (Supplementary Table S6). Based on inverse distance weight (IDW) analysis, the spatial variations in CNPS genes were investigated. We found that the C-degradation, C-fixation, C-methanol and P-cycling genes of native rhizosphere soils displayed similar spatial distribution patterns, with higher abundance in the northern region than in the southern region. This pattern was observed only in C-degradation genes of *A*. *palmeri* (Figure 3). This finding was further supported by the significance test in Supplementary Figure S4.

**Figure 4:**
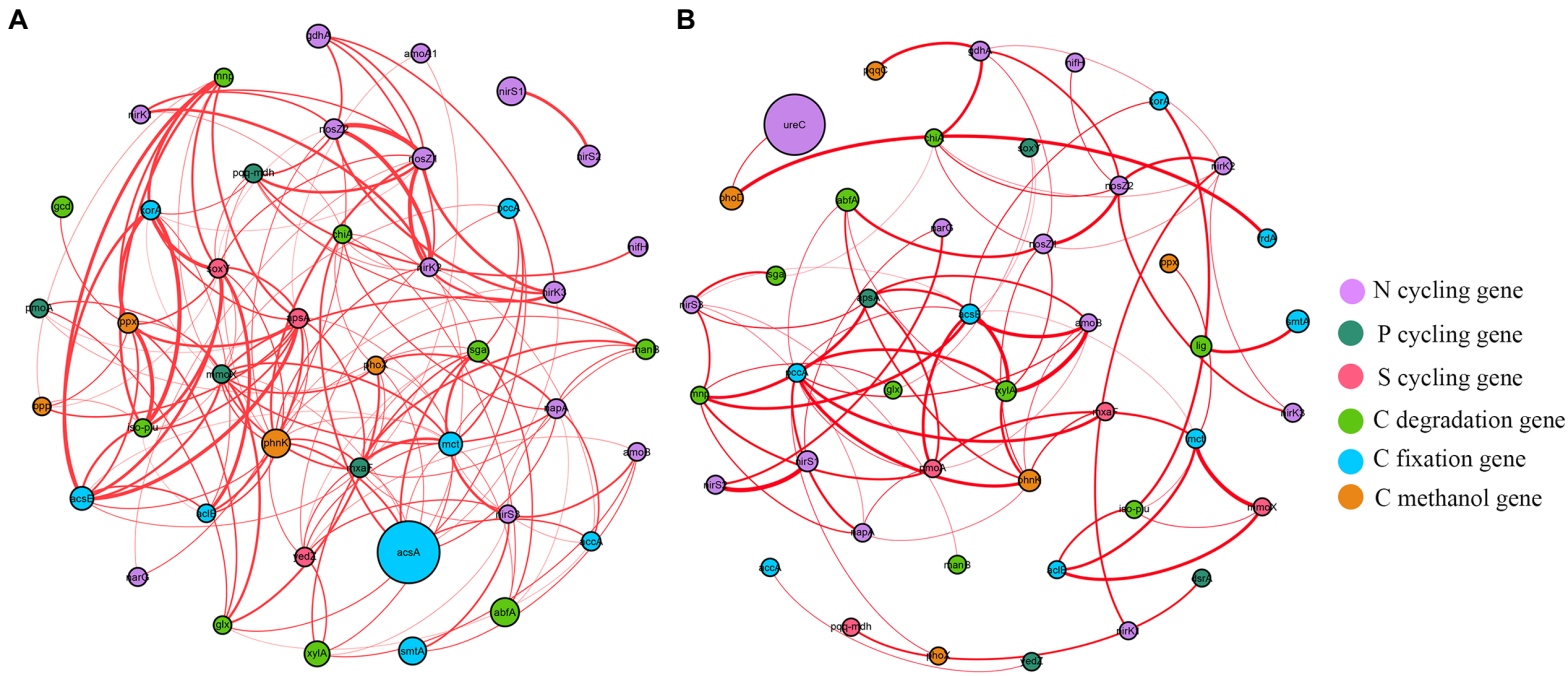
The co-occurrence network visualization of functional genes (R > 0.8 and *p* < 0.05) in *A*. *palmeri* **(A)** and native **(B)** rhizosphere soils. Nodes indicate functional genes. The size of each node is proportional to the relative abundance of each gene. And the thickness of each connection between two nodes (that is, edge) is proportional to the value of Spearman’s correlation coefficients. Edge color represents positive (red) correlation.

Functional molecular ecological networks (fMENs) of *A*. *palmeri* rhizosphere soils had 42 nodes and 154 edges, which was much higher than the 75 edges of native rhizosphere soils. All nodes in the *A*. *palmeri* and native soil networks were positively correlated with one another (Figure 4). The functional network topologies of *A*. *palmeri* and native rhizosphere soils were markedly different. The average degree and clustering coefficient were higher in *A*. *palmeri* soils than in native soils. In contrast, the distance and diameter were lower for the *A*. *palmeri* network (Supplementary Table S7).

### 3.3. Keystone taxa of rhizosphere soils

**Figure 5:**
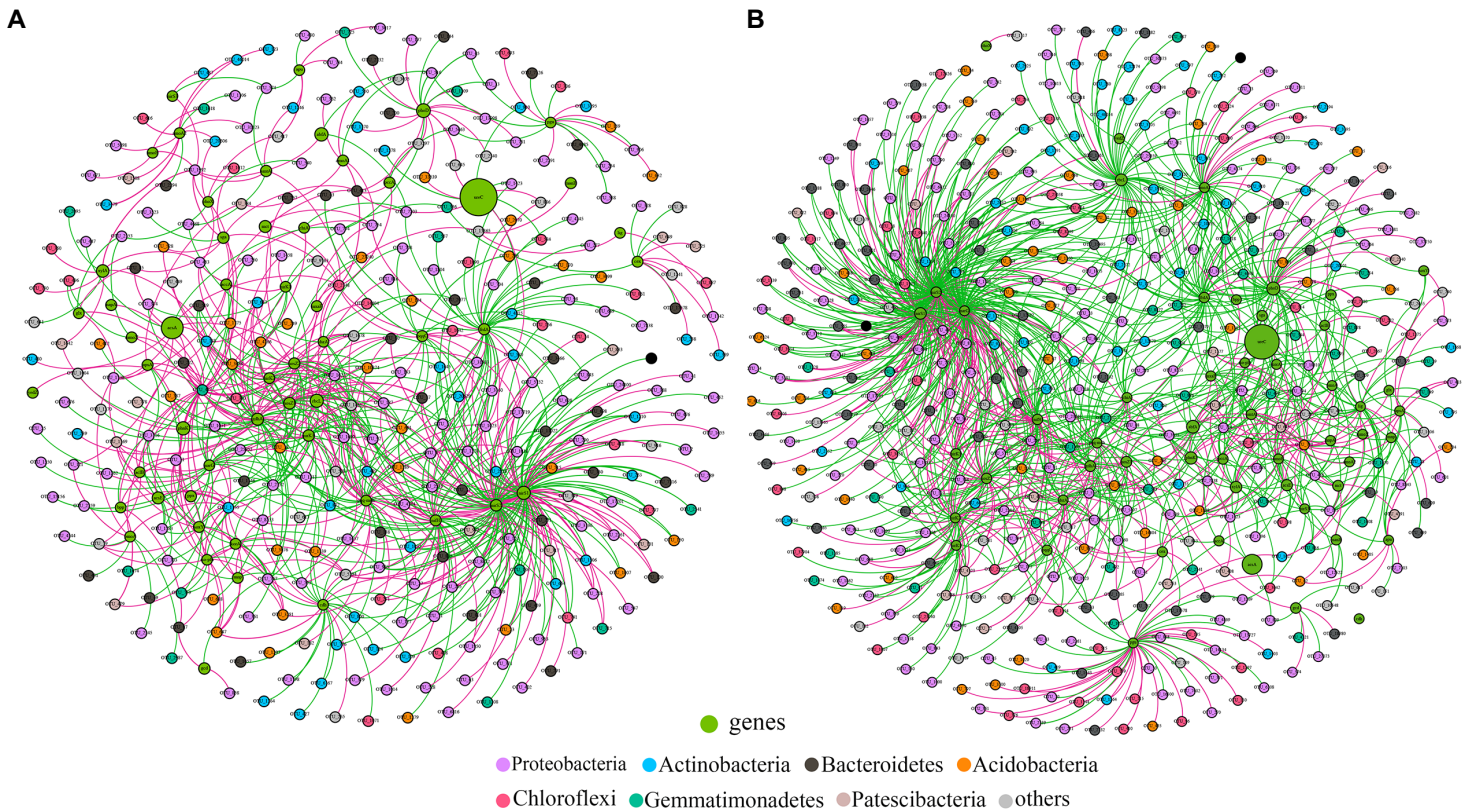
Functional molecular ecological networks between functional genes and bacterial OTUs in *A*. *palmeri* **(A)** and native **(B)** rhizosphere soils. The OTU nodes are colored according to taxonomy at the phylum level. Nodes in green are functional genes. Node size is proportional to its abundance. The red and green edges indicate the positive and negative correlations, respectively.

Co-occurrence networks of bacterial OTUs and functional genes were contrasted to explore the potential links between the bacterial community and functional capability (Figure 5). The top 5 node OTUs were considered as keystone taxa. Ultimately, OTU_437 (*Longimicrobiales*), OTU_1155 (*Kineosporiales*), OTU_1461 (*Armatimonadales*), OTU_1690 (*Rhizobiales*) and OTU_2132 (*Myxococcales*) were selected as keystone taxa in *A*. *palmeri* rhizosphere soils. It is noteworthy that the five keystone selected in *A*. *palmeri* rhizosphere soils were rare taxa, with an average abundance ranging from 6.3 × 10^−5^ to 3.24 × 10^−4^. In native rhizosphere soils, OTU_2751 (*Gemmatimonadales*), OTU_3 (*Sphingomonadales*), OTU_394 (*Sphingomonadales*), OTU_704 (unclassified order, Proteobacteria) and OTU_730 (*SBR1031*) were recognized as keystone OTUs (Table 1).

**Table 1:** Keystone OTUs in *A*. *palmeri* (AP) and native (N) rhizosphere soils.

AP	Keystones	Average abundance	Phylum	Class	Order	Family	Genus	Species
	OTU_437	0.000535	Gemmatimonadetes	Longimicrobia	Longimicrobiales	Longimicrobiaceae	—	—
	OTU_1155	0.000073	Actinobacteria	Actinobacteria	Kineosporiales	Kineosporiaceae	*Kineococcus*	*Kineococcus*_*endophyticus*
	OTU_1461	0.000063	Armatimonadetes	Armatimonadia	Armatimonadales	—	—	—
	OTU_1690	0.000324	Proteobacteria	Alphaproteobacteria	Rhizobiales	Xanthobacteraceae	*Rhodoplanes*	uncultured_*Rhodoplanes*_*sp*.
	OTU_2132	0.000102	Proteobacteria	Deltaproteobacteria	Myxococcales	Haliangiaceae	*Haliangium*	uncultured_bacterium
N	Keystones	Average abundance	Phylum	Class	Order	Family	Genus	Species
	OTU_2751	0.000121	Gemmatimonadetes	Gemmatimonadetes	Gemmatimonadales	Gemmatimonadaceae	*Gemmatirosa*	uncultured_bacterium
	OTU_3	0.025619	Proteobacteria	Alphaproteobacteria	Sphingomonadales	Sphingomonadaceae	*Sphingomonas*	—
	OTU_394	0.001522	Proteobacteria	Alphaproteobacteria	Sphingomonadales	Sphingomonadaceae	*Sphingomonas*	uncultured_bacterium
	OTU_730	0.000150	Chloroflexi	Anaerolineae	SBR1031	—	—	—
	OTU_704	0.000087	Proteobacteria	Alphaproteobacteria	—	—	—	—

### 3.4. Drivers of soil functional genes

**Figure 6:**
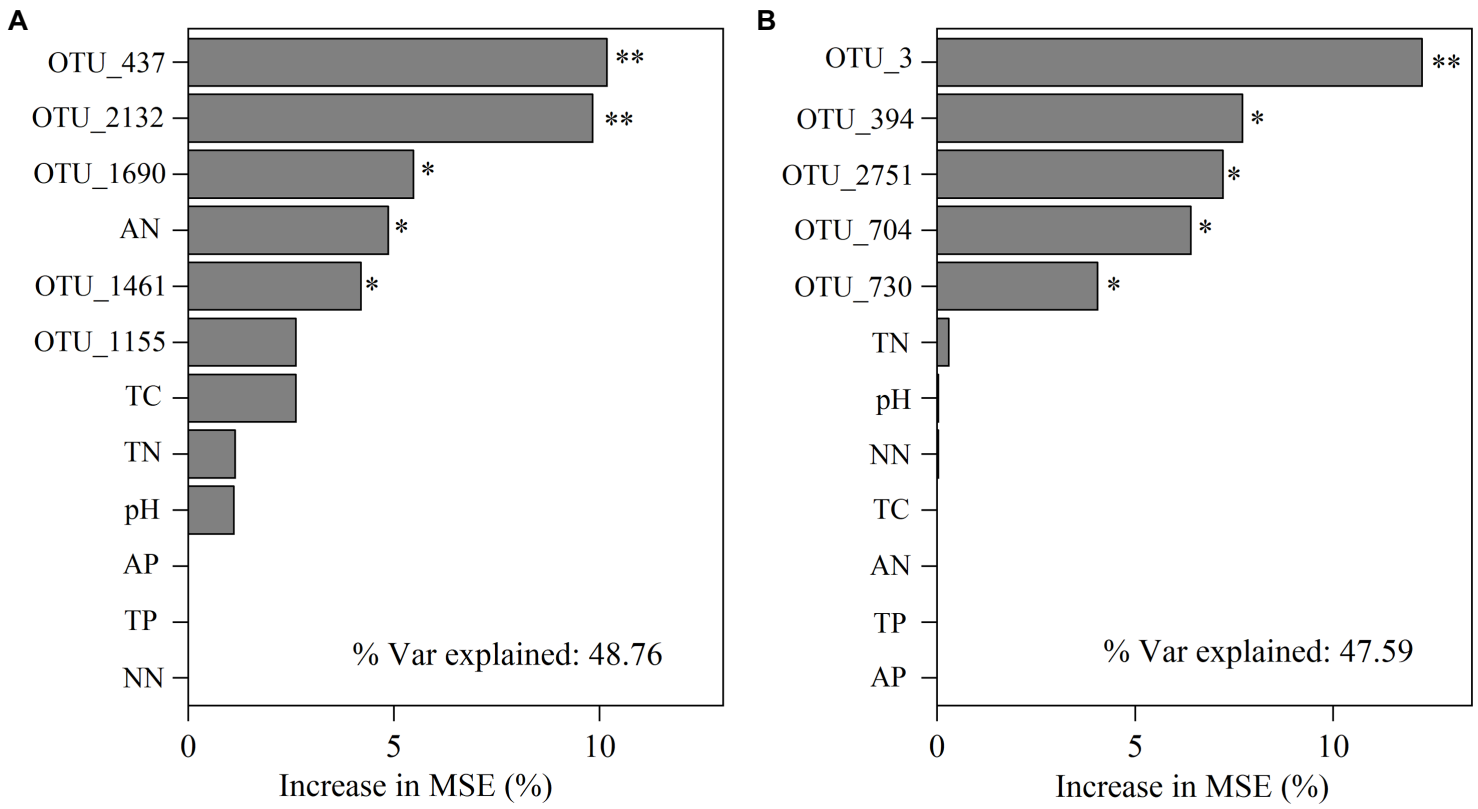
Mean predictor importance of the relative abundances of keystone taxa and edaphic factors on microbial functions of *A*. *palmeri* **(A)** and native **(B)** rhizosphere soils. The contribution (% of increased mean square error) is calculated based on random forest analyses. Higher MSE% values imply more important predictors (* *p* < 0.05, **p < 0.01). TN, total nitrogen; TC, total carbon; TP, total phosphorus; AN, ammonium nitrogen; NN, nitrate nitrogen; AP, available phosphorus.

**Figure 7:**
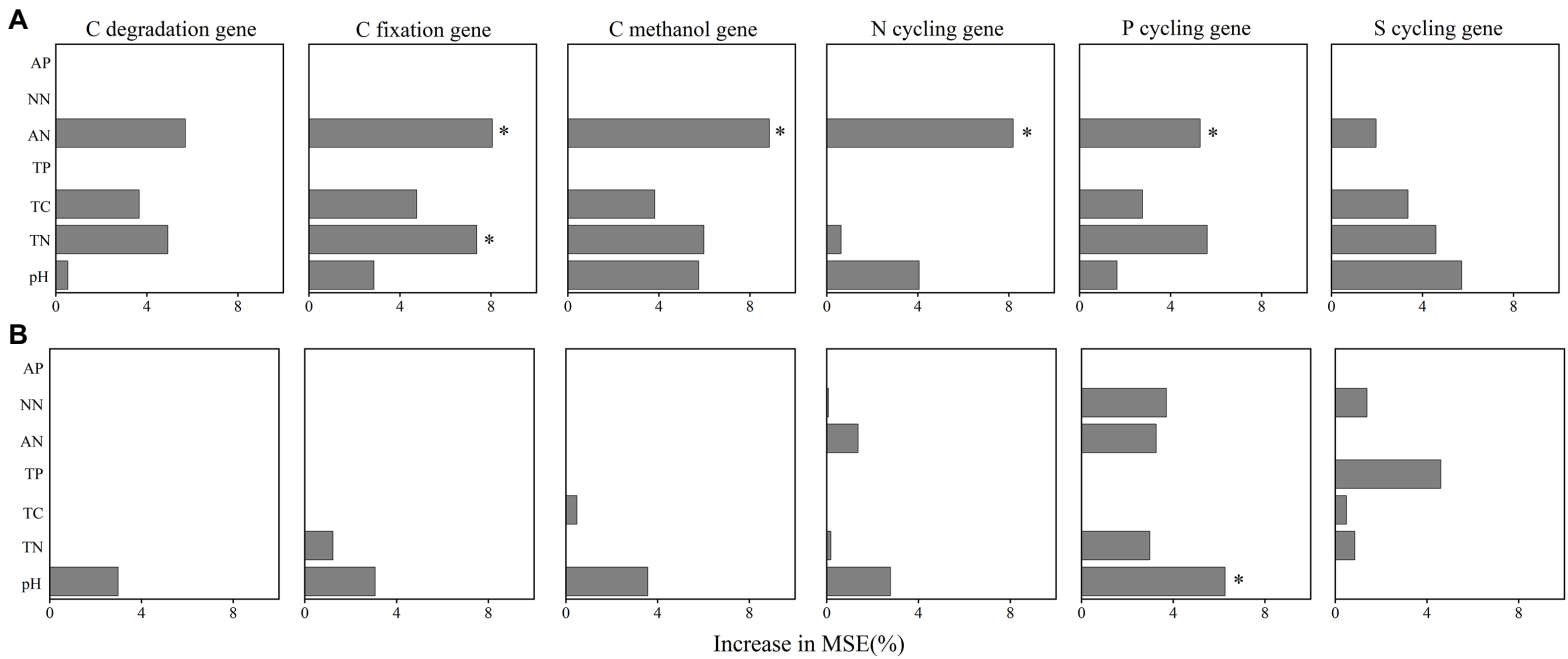
The importance of edaphic variables in explaining the variations functional genes’ abundances in *A*. *palmeri* **(A)** and native **(B)** rhizosphere soils. Random forest was used to determine the variable importance. Percentage increases in the MSE (mean squared error) of variables were used to estimate the importance of these predictors, and higher MSE% values imply more important predictors. Significance levels are as follows: *p < 0.05. MSE, mean squared error. The redundancy analysis (RDA) of edaphic variables on functional gene structure in *A*. *palmeri* **(A)** and native **(B)** rhizosphere soils. * *p* < 0.05, **p < 0.01). TN, total nitrogen; TC, total carbon; TP, total phosphorus; AN, ammonium nitrogen; NN, nitrate nitrogen; AP, available phosphorus.

We further performed random forest analysis to reveal the most important predictors (with a higher value of increase in MSE) for soil functional gene profiles. The results showed that keystone taxa played a stronger role in predicting gene profiles than physicochemical properties did (Figures 6A,B). Besides, more than 60% of the functional genes were positively or significantly positively linked to the relative abundance of the five keystone taxa in *A*. *palmeri* rhizosphere soils (Supplementary Table S8). In contrast, more than 80% of the functional genes were negatively or significantly negatively correlated with relative abundance of the five keystone OTUs in native rhizosphere soils (Supplementary Table S9). The relative abundance of keystone within *A*. *palmeri* rhizosphere soils presented significant positive correlations with the abundance of functional genes (*R^2^* = 0.19, *p* = 0.04, Supplementary Figure S5A), while there were significant negative associations between keystone and functional genes within native rhizosphere soils (*R^2^* = 0.60, *p* < 0.01, Supplementary Figure S5B). This result was also reflected in network analysis of bacterial OTUs and functional genes, with a drastic increase of negative edges from 378 in *A*. *palmeri* rhizosphere soils to 878 in native rhizosphere soils (Figure 5; Supplementary Table S10). Among the soil physicochemical parameters, ammonium nitrogen was the major predictor driving the variations of functional genes, especially carbon-degradation, carbon-fixation, carbon-methanol, nitrogen-cycling and phosphorus-cycling genes (*p* < 0.05) in *A*. *palmeri* rhizosphere soils (Figures 6A, 7A). However, pH was the most important factor for phosphorus-cycling genes (*p* < 0.05) in native rhizosphere soils (Figure 7B).

## 4. Discussion

### 4.1. Bacterial community respond to *Amaranthus palmeri* invasion

Different vegetation recruits different soil microbes, creating a variety of microhabitats. Our research revealed that the predominant phyla were Proteobacteria, Bacteroidetes, Acidobacteria and Actinobacteria. These bacterial taxa are prevalent in numerous terrestrial habitats and are widely dispersed around the world (Bai et al., 2017; Wolińska et al., 2017; Chen Q. L. et al., 2020). We noticed that the rhizosphere soils of *A*. *palmeri* contained more Bacteroidetes and Nitrospirae and less Actinobacteria than in native plants. Bacteroidetes are found in many different habitats and are crucial to the turnover of organic matter. In order to facilitate the nitrogen cycle in soil, Mohan et al. (2004) proposed that Bacteroidetes could mediate nitrate reduction to ammonium. Additionally, Bacteroidetes have the ability to degrade chitin and cellulose (Trivedi et al., 2013). Delgado-Baquerizo et al. (2017) emphasized the significance of Bacteroidetes in controlling the breakdown of organic matter and the cycling of carbon in soils. The observable alterations in Nitrospirae appeared to be caused by the large increase in the abundance of the Nitrospiraceae family (Supplementary Table S5). The Nitrospiraceae family is known to support important soil processes, with some members participating in soil nitrification (Chen L. et al., 2020; Delgado-Baquerizo et al., 2020; Vuko et al., 2020). Actinobacteria, known as copiotrophic bacteria, prefer to live on abundant resources (Ramirez et al., 2012; Ding et al., 2015b). This outcome was in line with the variations in soil properties (Supplementary Table S11).

### 4.2. Effect of *Amaranthus palmeri* invasion on keystone species

Keystone taxa are highly linked groups that are essential to the upkeep of complex networks (Berry and Widder, 2014; Ma et al., 2016; Herren and McMahon, 2018). Five keystone taxa were found in the rhizosphere soils of *A*. *palmeri*, including the unclassified family Longimicrobiaceae, *Kineococcus endophyticus* (Kineosporiaceae), unclassified order Armatimonadales, uncultured_*Rhodoplanes*_*sp*. (Xanthobacteraceae) and uncultured_bacterium (*Haliangium*). *Kineococcus endophyticus* (OTU_1155), the unclassified family Longimicrobiaceae (OTU_437), and the unclassified order Armatimonadales (OTU_1461) were found to have positive or significant positive associations with the relative abundance of 52 functional genes (Supplementary Table S8). Longimicrobiaceae (Gemmatimonadetes) has been found in Mediterranean forest soils. Members of the Longimicrobiaceae family can help with phosphate dissolution in soils. Some bacterial groups in this family can survive in harsh environments, and they are commonly found in semiarid soils with little organic matter (Korkar et al., 2022; Wu et al., 2022). *Kineococcus endophyticus* (Actinobacteria) can synthetize the plant hormone indole-3-acetic acid (IAA), and some IAA’s effects can encourage root lengthening and the number of root branches and hairs, which may enable host plants to have more capacity for absorbing moisture and nutrients. In addition, *Kineococcus* has a high level of metal tolerance (e.g., Zn, Cu, etc.) (Vannucchi et al., 2021). The members of the genera *Sphingomonas* and *Gemmatirosa* were categorized as keystone taxa in native rhizosphere soils. *Gemmatirosa* play important roles in mediating P cycling, The genera of *Sphingomonas* are major degraders of herbicide and polycyclic aromatic hydrocarbons (Guo et al., 2011; Jiao et al., 2019; Liu et al., 2022). Our findings highlighted the importance of keystone taxa in multiple communities, the alterations in keystone taxa may play essential roles in the survival and success of *A*. *palmeri*.

### 4.3. Cooccurrence patterns of functional genes

Changes in microbial communities could have a dramatic impact on the C, N, S, and P metabolic cycles that they mediate (Banerjee et al., 2019; Fan et al., 2021). However, we still do not know if and how plant invasion affects the complexity of interconnection among cooccurring functional networks. Previous studies have mostly concentrated on the α and β diversity patterns of invasive plants on soil microorganisms. Our findings showed that cooccurrence networks of functional genes varied between invasive and native rhizosphere soils (Figure 4; Supplementary Table S7). The positive linkages increased from 75 in the native network to 154 in the *A*. *palmeri* network, showing greater cooperation of microbial functional genes as a result of plant invasion. The topological features of the *A*. *palmeri* network had higher average degree and clustering coefficient, and lower average distance and diameters, reflecting more potential complexity of soil functional networks (Ma et al., 2016; Jiao et al., 2022). The enhancement of network complexity and positive linkages under *A*. *palmeri*-invaded soils with respect to native soils might be an advantageous response of the soil microbiome to environmental changes (Santolini and Barabási, 2018; Jiao et al., 2022). The changes in topological attributes may be combined with the replacement of keystone OTUs (Lin et al., 2021). For instance, the unclassified family Longimicrobiaceae (OTU_437) had a net degree of up to 27, which was strongly connected to more than half of the CNPS-cycling genes found in our analysis (Figure 5; Supplementary Table S12).

### 4.4. Drivers of soil functions

The effects of plant invasion on local vegetation and microbial community in the soil are well documented (Mamet et al., 2017; Wang et al., 2018; Shen et al., 2021; Mo et al., 2022). However, there is no empirical evidence to support the relative importance of soil edaphic characteristics vs. keystone taxa in regulating microbial functional processes involved in CNPS cycling. Predictions of complex, dynamic microbial networks enable us to establish mechanistic models connecting community composition, edaphic features, and functional properties in metagenomic datasets (Chen et al., 2019; Mamet et al., 2019). We found that the keystone taxa best explained the functional genes with the explanation of 32.27 and 37.61% in *A*. *palmeri* rhizosphere soils and native rhizosphere soils, respectively (Figures 6A,B). This outcome corroborated previous findings that keystone taxa play critical roles in controlling soil microbial functional capacity (Zhang et al., 2019; Shi et al., 2020; Tian et al., 2021). Besides, we further found that the keystone taxa in *A*. *palmeri* rhizosphere soils were significantly positively correlated with the functional genes of soil microbial communities compared with native plants (Supplementary Figure S5A). Therefore, changes in keystone taxa due to plant invasion may be related to functional alterations in the soil microbial community. Our finding highlighted the importance of keystone taxa in functional stability in the invaded ecosystems.

The indicators driving the soil microbial functions differed between *A*. *palmeri* and native plants. For instance, significant driving factors of soil N-NH_4_ on the function profiles of *A*. *palmeri* were found based on random forest analysis (Figure 6A). Besides, N-NH_4_ was identified as the major driver for the variation of C-degradation, C-fixation, C-methanol, N-cycling and P-cycling genes (Figure 7A). The abundances of these genes also had a significantly negative relationship with N-NH_4_ (Supplementary Figure S7), which is consistent with our earlier research suggesting that *A*. *palmeri* may prefer to absorb and utilize N-NH_4_ (Zhang M. et al., 2022). These findings demonstrated that invasive plants impacted the soil nutrient status and fundamentally altered ecosystem processes (Zhao et al., 2019). The pH effect on bacterial communities is well documented (Pan et al., 2014; Hill et al., 2016; Cheng et al., 2021). Under conditions of plant invasion, keystone species that were insensitive or less sensitive to pH were replaced by species that were sensitive to pH (Supplementary Figure S6), and the invasion of *A*. *palmeri* shaped keystone species, resulting in the development of environmental adaptation patterns that were distinct from those of native communities (Lin et al., 2021). Our study provides a theoretical underpinning and support for the prevention and management of invasive *A*. *palmeri*. For instance, adding organic fertilizer or lime to the soil to regulate its pH or altering other soil qualities to modify the functions of the invaded ecosystems’ soils can make the environment unsuitable for the flourishing of *A*. *palmeri*.

## 5. Conclusion

In conclusion, *A*. *palmeri* distinctly altered the structure and functional potential of the soil microbial community. *A*. *palmeri* had higher levels of Bacteroidetes and Nitrospirae and lower levels of Actinobacteria than native plants. The complexity of co-occurrence networks of soil functional genes was increased by *A*. *palmeri* invasion. The spatial distribution patterns of C degradation, C fixation, C methanol, N cycling, P cycling and S cycling genes differed between *A*. *palmeri* and native rhizosphere soils. The edaphic characteristics were less important in controlling the abundance of microbial functional genes than the keystone taxa. The relative abundance of keystone within *A*. *palmeri* rhizosphere soils demonstrated significant positive correlations with the abundance of functional genes, indicating that keystone taxa played important ecological roles in supporting soil function in the invaded ecosystems.

## Data availability statement

The data presented in the study are deposited in the Sequence Reader Archive repository, accession number PRJNA 945232.

## Author contributions

MZ and FS made a conceptualization of the study. MZ, XL, and KW conducted the field work. MZ, CS, KW, and ZQ collected the data. MZ wrote the manuscript. CS and FS supervised and reviewed the manuscript. All authors contributed to the final version of the manuscript.

## Funding

This research was financially supported by the project of Tianjin Municipal Education Commission, Tianjin, China (No. 2020YJSB121).

## Conflict of interest

The authors declare that the research was conducted in the absence of any commercial or financial relationships that could be construed as a potential conflict of interest.

## Publisher’s note

All claims expressed in this article are solely those of the authors and do not necessarily represent those of their affiliated organizations, or those of the publisher, the editors and the reviewers. Any product that may be evaluated in this article, or claim that may be made by its manufacturer, is not guaranteed or endorsed by the publisher.

## Footnotes

^1^ http://www.R-project.org/

## References

[ref1] Anthony M. A. Stinson K. A. Moore J. A. Frey S. D. (2020). Plant invasion impacts on fungal community structure and function depend on soil warming and nitrogen enrichment. Oecologia 194, 659–672. doi: 10.1007/s00442-020-04797-4 33141324 PMC7683454

[ref2] Bai R. Wang J. T. Deng Y. He J. Z. Feng K. Zhang L. M. (2017). Microbial community and functional structure significantly varied among distinct types of paddy soils but responded differently along gradients of soil depth layers. Front. Microbiol. 8:945. doi: 10.3389/fmicb.2017.00945 28611747 PMC5447084

[ref3] Banerjee S. Walder F. Büchi L. Meyer M. Held A. Y. Gattinger A.. (2019). Agricultural intensification reduces microbial network complexity and the abundance of keystone taxa in roots. ISME J. 13, 1722–1736. doi: 10.1038/s41396-019-0383-2 30850707 PMC6591126

[ref4] Bastian M. Heymann S. Jacomy M. (2009). Gephi: an open source software for exploring and manipulating networks. In Proceedings of the International AAAI Conference on Web and Social Media.

[ref5] Berry D. Widder S. (2014). Deciphering microbial interactions and detecting keystone species with co-occurrence networks. Front. Microbiol. 5:219. doi: 10.3389/fmicb.2014.00219 24904535 PMC4033041

[ref6] Breiman L. (2001). Random forests. Mach. Learn. 45, 5–32. doi: 10.1023/A:1010933404324

[ref7] Chen Q. L. Ding J. Li C. Y. Yan Z. Z. He J. Z. Hu H. W. (2020). Microbial functional attributes, rather than taxonomic attributes, drive top soil respiration, nitrification and denitrification processes. Sci. Total Environ. 734:139479. doi: 10.1016/j.scitotenv.2020.139479 32464393

[ref8] Chen L. J. Jiang Y. J. Liang C. Luo Y. Xu Q. S. Han C.. (2019). Competitive interaction with keystone taxa induced negative priming under biochar amendments. Microbiome. 7, 77–18. doi: 10.1186/s40168-019-0693-7 31109381 PMC6526607

[ref9] Chen L. Li F. Li W. Ning Q. Li J. W. Zhang J. B.. (2020). Organic amendment mitigates the negative impacts of mineral fertilization on bacterial communities in Shajiang black soil. Appl. Soil Ecol. 150:103457. doi: 10.1016/j.apsoil.2019.103457

[ref10] Cheng J. M. Han Z. J. Cong J. Yu J. J. Zhou J. Z. Zhao M. X.. (2021). Edaphic variables are better indicators of soil microbial functional structure than plant-related ones in subtropical broad-leaved forests. Sci. Total Environ. 773:145630. doi: 10.1016/j.scitotenv.2021.145630 33582323

[ref11] Cheng Z. Y. Shi J. C. He Y. Wu L. S. Xu J. M. (2022). Assembly of root-associated bacterial community in cadmium contaminated soil following five-year consecutive application of soil amendments: evidences for improved soil health. J. Hazard. Mater. 426:128095. doi: 10.1016/j.jhazmat.2021.128095 34952504

[ref12] Cordero O. X. Datta M. S. (2016). Microbial interactions and community assembly at microscales. Curr. Opin. Microbiol. 31, 227–234. doi: 10.1016/j.mib.2016.03.015 27232202 PMC5157693

[ref13] Craig M. E. Pearson S. M. Fraterrigo J. M. (2015). Grass invasion effects on forest soil carbon depend on landscape-level land use patterns. Ecology 96, 2265–2279. doi: 10.1890/14-1770.1 26405751

[ref14] Cui B. Liu X. H. Yang Q. Li J. M. Zhou X. Y. Peng Y. Z. (2017). Achieving partial denitrification through control of biofilm structure during biofilm growth in denitrifying biofilter. Bioresour. Technol. 238, 223–231. doi: 10.1016/j.biortech.2017.04.034 28433912

[ref15] Delgado-Baquerizo M. Reich P. B. Trivedi C. Eldridge D. J. Abades S. Alfaro F. D.. (2020). Multiple elements of soil biodiversity drive ecosystem functions across biomes. Nat. Ecol. Evol. 4, 210–220. doi: 10.1038/s41559-019-1084-y 32015427

[ref16] Delgado-Baquerizo M. Trivedi P. Trivedi C. Eldridge D. J. Reich P. B. Jeffries T. C.. (2017). Microbial richness and composition independently drive soil multifunctionality. Funct. Ecol. 31, 2330–2343. doi: 10.1111/1365-2435.12924

[ref17] Ding J. J. Zhang Y. G. Deng Y. Cong J. Lu H. Sun X.. (2015a). Integrated metagenomics and network analysis of soil microbial community of the forest timberline. Sci. Rep. 5:7994. doi: 10.1038/srep07994 25613225 PMC4303876

[ref18] Ding J. J. Zhang Y. G. Wang M. M. Sun X. Cong J. Deng Y.. (2015b). Soil organic matter quantity and quality shape microbial community compositions of subtropical broadleaved forests. Mol. Ecol. 24, 5175–5185. doi: 10.1111/mec.13384 26363284

[ref19] Edgar R. C. (2013). UPARSE: highly accurate OTU sequences from microbial amplicon reads. Nat. Methods 10, 996–998. doi: 10.1038/nmeth.2604 23955772

[ref20] Fan K. K. Delgado-Baquerizo M. Guo X. S. Wang D. Z. Zhu Y. G. Chu H. Y. (2021). Biodiversity of key-stone phylotypes determines crop production in a 4-decade fertilization experiment. ISME J. 15, 550–561. doi: 10.1038/s41396-020-00796-8 33028975 PMC8027226

[ref21] Geng P. X. Ma A. Z. Wei X. X. Chen X. K. Yin J. Hu F. T.. (2022). Interaction and spatio-taxonomic patterns of the soil microbiome around oil production wells impacted by petroleum hydrocarbons. Environ. Pollut. 307:119531. doi: 10.1016/j.envpol.2022.119531 35623572

[ref22] Guo C. L. Ke L. Dang Z. Tam N. F. (2011). Temporal changes in Sphingomonas and mycobacterium populations in mangrove sediments contaminated with different concentrations of polycyclic aromatic hydrocarbons (PAHs). Mar. Pollut. Bull. 62, 133–139. doi: 10.1016/j.marpolbul.2010.08.022 20926106

[ref23] Herren C. M. McMahon K. D. (2018). Keystone taxa predict compositional change in microbial communities. Environ. Microbiol. 20, 2207–2217. doi: 10.1111/1462-2920.14257 29708645

[ref24] Hill R. Saetnan E. R. Scullion J. Gwynn-Jones D. Ostle N. Edwards A. (2016). Temporal and spatial influences incur reconfiguration of Arctic heathland soil bacterial community structure. Environ. Microbiol. 18, 1942–1953. doi: 10.1111/1462-2920.13017 26259508

[ref25] Huang J. X. Xu X. Wang M. Nie M. Qiu S. Y. Wang Q.. (2016). Responses of soil nitrogen fixation to Spartina alterniflora invasion and nitrogen addition in a Chinese salt marsh. Sci. Rep. 6, 1–8. doi: 10.1038/srep20384 26869197 PMC4751540

[ref26] Jiao S. Chen W. M. Wei G. H. (2022). Core microbiota drive functional stability of soil microbiome in reforestation ecosystems. Glob. Chang. Biol. 28, 1038–1047. doi: 10.1111/gcb.16024 34862696

[ref27] Jiao S. Xu Y. Q. Zhang J. Hao X. Lu Y. H. (2019). Core microbiota in agricultural soils and their potential associations with nutrient cycling. mSystems 4, e00313–e00318. doi: 10.1128/mSystems.00313-18 30944882 PMC6435817

[ref28] Jin H. Chang L. van Kleunen M. Liu Y. J. (2022). Soil mesofauna may buffer the negative effects of drought on alien plant invasion. J. Ecol. 110, 2332–2342. doi: 10.1111/1365-2745.13950

[ref29] Kamutando C. N. Vikram S. Kamgan-Nkuekam G. Makhalanyane T. P. Greve M. le Roux J. J.. (2019). The functional potential of the rhizospheric microbiome of an invasive tree species Acacia dealbata. Microb. Ecol. 77, 191–200. doi: 10.1007/s00248-018-1214-0 29948018

[ref30] Korkar M. H. Magdy M. Rizk S. M. Fiteha Y. G. Atta A. H. Rashed M. A. S. (2022). Rhizosphere-associated, microbiome profile of agriculture reclaimed lands in Egypt. Preprints. doi: 10.20944/preprints202204.0265.v1,

[ref31] Lin W. C. Lu J. Q. Yao H. Y. Lu Z. B. He Y. M. Mu C. K.. (2021). Elevated pCO_2_ alters the interaction patterns and functional potentials of rearing seawater microbiota. Environ. Pollut. 287:117615. doi: 10.1016/j.envpol.2021.117615 34171732

[ref32] Linders T. E. W. Schaffner U. Eschen R. Abebe A. Choge S. K. Nigatu L.. (2019). Direct and indirect effects of invasive species: biodiversity loss is a major mechanism by which an invasive tree affects ecosystem functioning. J. Ecol. 107, 2660–2672. doi: 10.1111/1365-2745.13268

[ref33] Liu X. Q. Liu H. R. Ren D. Y. Liu C. R. Zhang Y. S. Wang S. Q.. (2022). Interlinkages between soil properties and keystone taxa under different tillage practices on the North China plain. Appl. Soil Ecol. 178:104551. doi: 10.1016/j.apsoil.2022.104551

[ref34] Lynn T. M. Ge T. D. Yuan H. Z. Wei X. M. Wu X. H. Xiao K. Q.. (2017). Soil carbon-fixation rates and associated bacterial diversity and abundance in three natural ecosystems. Microb. Ecol. 73, 645–657. doi: 10.1007/s00248-016-0890-x 27838764

[ref35] Ma B. Wang H. Z. Dsouza M. Lou J. He Y. Dai Z. M.. (2016). Geographic patterns of co-occurrence network topological features for soil microbiota at continental scale in eastern China. ISME J. 10, 1891–1901. doi: 10.1038/ismej.2015.261 26771927 PMC5029158

[ref36] Mamet S. D. Lamb E. G. Piper C. L. Winsley T. Siciliano S. D. (2017). Archaea and bacteria mediate the effects of native species root loss on fungi during plant invasion. ISME J. 11, 1261–1275. doi: 10.1038/ismej.2016.205 28140393 PMC5437935

[ref37] Mamet S. D. Redlick E. Brabant M. Lamb E. G. Helgason B. L. Stanley K.. (2019). Structural equation modeling of a winnowed soil microbiome identifies how invasive plants re-structure microbial networks. ISME J. 13, 1988–1996. doi: 10.1038/s41396-019-0407-y 30926920 PMC6776034

[ref38] Mo S. M. He S. Sang Y. M. Li J. H. Kashif M. Zhang Z. F.. (2022). Integration of microbial transformation mechanism of polyphosphate accumulation and sulfur cycle in subtropical marine mangrove ecosystems with Spartina alterniflora invasion. Microb. Ecol. 85, 478–494. doi: 10.1007/s00248-022-01979-w 35157108

[ref39] Mohan S. B. Schmid M. Jetten M. Cole J. (2004). Detection and widespread distribution of the nrfA gene encoding nitrite reduction to ammonia, a short circuit in the biological nitrogen cycle that competes with denitrification. FEMS Microbiol. Ecol. 49, 433–443. doi: 10.1016/j.femsec.2004.04.012 19712292

[ref40] Nasto M. K. McLeod M. L. Bullington L. Lekberg Y. Stark J. M. (2022). The effect of plant invasion on soil microbial carbon-use efficiency in semi-arid grasslands of the Rocky Mountain west. J. Ecol. 110, 479–493. doi: 10.1111/1365-2745.13815

[ref41] Pan Y. Cassman N. de Hollander M. Mendes L. W. Korevaar H. Geerts R. H.. (2014). Impact of long-term N, P, K, and NPK fertilization on the composition and potential functions of the bacterial community in grassland soil. FEMS Microbiol. Ecol. 90, 195–205. doi: 10.1111/1574-6941.12384 25046442

[ref700] R Core Team (2013). R: A Language And Environment For Statistical Computing. Vienna: R Foundation for Statistical Computing.

[ref42] Ramirez K. S. Craine J. M. Fierer N. (2012). Consistent effects of nitrogen amendments on soil microbial communities and processes across biomes. Glob. Chang. Biol. 18, 1918–1927. doi: 10.1111/j.1365-2486.2012.02639.x

[ref43] Rodríguez-Caballero G. Caravaca F. Alguacil M. M. Fernández-López M. Fernández-González A. J. Roldán A. (2017). Striking alterations in the soil bacterial community structure and functioning of the biological N cycle induced by Pennisetum setaceum invasion in a semiarid environment. Soil Biol. Biochem. 109, 176–187. doi: 10.1016/j.soilbio.2017.02.012

[ref44] Santolini M. Barabási A. L. (2018). Predicting perturbation patterns from the topology of biological networks. PNAS Nexus 115, E6375–E6383. doi: 10.1073/pnas.1720589115 PMC6142275 29925605

[ref45] Seebens H. Essl F. Dawson W. Fuentes N. Moser D. Pergl J.. (2015). GlobCite al trade will accelerate plant invasions in emerging economies under climate change. Glob. Chang. Biol. 21, 4128–4140. doi: 10.1111/gcb.13021 26152518

[ref46] Shannon-Firestone S. Reynolds H. L. Phillips R. P. Flory S. L. Yannarell A. (2015). The role of ammonium oxidizing communities in mediating effects of an invasive plant on soil nitrification. Soil Biol. Biochem. 90, 266–274. doi: 10.1016/j.soilbio.2015.07.017

[ref47] Shen C. C. Wang J. He J. Z. Yu F. H. Ge Y. (2021). Plant diversity enhances soil fungal diversity and microbial resistance to plant invasion. Appl. Environ. Microb. 87, e00251–e00221. doi: 10.1128/AEM.00251-21 PMC8208136 33741636

[ref48] Shi Y. Delgado-Baquerizo M. Li Y. T. Yang Y. F. Zhu Y. G. Peñuelas J.. (2020). Abundance of kinless hubs within soil microbial networks are associated with high functional potential in agricultural ecosystems. Environ. Int. 142:105869. doi: 10.1016/j.envint.2020.105869 32593837

[ref49] Sun F. Zeng L. D. Cai M. L. Chauvat M. Forey E. Tariq A.. (2022). An invasive and native plant differ in their effects on the soil food-web and plant-soil phosphorus cycle. Geoderma 410:115672. doi: 10.1016/j.geoderma.2021.115672

[ref50] Tian W. Xiang X. Wang H. (2021). Differential impacts of water table and aemperature on bacterial communities in pore water from a subalpine peatland, Central China. Front. Microbiol. 12:649981. doi: 10.3389/fmicb.2021.649981 34122363 PMC8193233

[ref51] Trivedi P. Anderson I. C. Singh B. K. (2013). Microbial modulators of soil carbon storage: integrating genomic and metabolic knowledge for global prediction. Trends Microbiol. 21, 641–651. doi: 10.1016/j.tim.2013.09.005 24139848

[ref52] Vannucchi F. Imperato V. Saran A. Staykov S. D’Haen J. Sebastiani L.. (2021). Inoculated seed endophytes modify the poplar responses to trace elements in polluted soil. Agronomy 11:1987. doi: 10.3390/agronomy11101987

[ref53] Vuko M. Cania B. Vogel C. Kublik S. Schloter M. Schulz S. (2020). Shifts in reclamation management strategies shape the role of exopolysaccharide and lipopolysaccharide-producing bacteria during soil formation. Microb. Biotechnol. 13, 584–598. doi: 10.1111/1751-7915.13532 31920012 PMC7017822

[ref54] Wang T. T. Hao Y. W. Zhu M. Z. Yu S. T. Ran W. Xue C.. (2019). Characterizing differences in microbial community composition and function between Fusarium wilt diseased and healthy soils under watermelon cultivation. Plant Soil 438, 421–433. doi: 10.1007/s11104-019-04037-6

[ref55] Wang C. Y. Jiang K. Zhou J. W. Wu B. D. (2018). Solidago canadensis invasion affects soil N-fixing bacterial communities in heterogeneous landscapes in urban ecosystems in East China. Sci. Total Environ. 631-632, 702–713. doi: 10.1016/j.scitotenv.2018.03.061 29544175

[ref56] Wang C. Wang W. Q. Sardans J. Ouyang L. M. Tong C. Asensio D.. (2020). Higher fluxes of C, N and P in plant/soil cycles associated with plant invasion in a subtropical estuarine wetland in China. Sci. Total Environ. 730:139124. doi: 10.1016/j.scitotenv.2020.139124 32388112

[ref57] Wolińska A. Kuźniar A. Zielenkiewicz U. Izak D. Szafranek-Nakonieczna A.. (2017). Bacteroidetes as a sensitive biological indicator of agricultural soil usage revealed by a culture-independent approach. Appl. Soil Ecol. 119, 128–137. doi: 10.1016/j.apsoil.2017.06.009

[ref58] Wu X. J. Rensing C. Han D. F. Xiao K. Q. Dai Y. X. Tang Z. X.. (2022). Genome-resolved metagenomics reveals distinct phosphorus acquisition strategies between soil microbiomes. Msystems 7:e0110721. doi: 10.1128/msystems.01107-21 35014868 PMC8751388

[ref59] Xiang Q. Chen Q. L. Zhu D. Yang X. R. Qiao M. Hu H. W.. (2020). Microbial functional traits in phyllosphere are more sensitive to anthropogenic disturbance than in soil. Environ. Pollut. 265:114954. doi: 10.1016/j.envpol.2020.114954 32544665

[ref60] Xu M. Hao X. L. Xiong Z. Q. Liao H. Wang L. Zhang T. Y.. (2021). Soil amendments change bacterial functional genes more than taxonomic structure in a cadmium-contaminated soil. Soil Biol. Biochem. 154:108126. doi: 10.1016/j.soilbio.2020.108126

[ref61] Xu H. W. Liu Q. Wang S. Y. Yang G. S. Xue S. (2022). A global meta-analysis of the impacts of exotic plant species invasion on plant diversity and soil properties. Sci. Total Environ. 810:152286. doi: 10.1016/j.scitotenv.2021.152286 34902405

[ref62] Yin L. J. Liu B. Wang H. C. Zhang Y. Wang S. Jiang F.. (2020). The rhizosphere microbiome of Mikania micrantha provides insight into adaptation and invasion. Front. Microbiol. 11:1462. doi: 10.3389/fmicb.2020.01462 32733410 PMC7359623

[ref63] Zhang G. L. Bai J. H. Tebbe C. C. Huang L. B. Jia J. Wang W.. (2022). Plant invasion reconstructs soil microbial assembly and functionality in coastal salt marshes. Mol. Ecol. 31, 4478–4494. doi: 10.1111/mec.16600 35789059

[ref64] Zhang K. R. Li X. S. Cheng X. L. Zhang Z. H. Zhang Q. F. (2019). Changes in soil properties rather than functional gene abundance control carbon and nitrogen mineralization rates during long-term natural revegetation. Plant Soil 443, 293–306. doi: 10.1007/s11104-019-04212-9

[ref65] Zhang M. Li X. Y. Qiu Z. L. Shi C. Wang K. F. Fukuda K.. (2022). Effects of Amaranthus palmeri invasion on soil extracellular enzyme activities and enzymatic stoichiometry. J. Soil Sci. Plant Nut. 22, 5183–5194. doi: 10.1007/s42729-022-00994-7

[ref66] Zhang M. Ma K. X. Liu T. Tang L. L. Khan A. A. Yang T.. (2020). Responses in phenotypic plasticity of Amaranthus palmeri and Polygonum orientale to soil factors under different habitats. Clean-Soil Air Water. 48:1900203. doi: 10.1002/clen.201900203

[ref67] Zhang Z. L. Suseela V. (2021). Nitrogen availability modulates the impacts of plant invasion on the chemical composition of soil organic matter. Soil Biol. Biochem. 156:108195. doi: 10.1016/j.soilbio.2021.108195

[ref68] Zhao M. X. Lu X. F. Zhao H. X. Yang Y. F. Hale L. Gao Q.. (2019). Ageratina adenophora invasions are associated with microbially mediated differences in biogeochemical cycles. Sci. Total Environ. 677, 47–56. doi: 10.1016/j.scitotenv.2019.04.330 31051382

[ref69] Zheng B. X. Zhu Y. G. Sardans J. Peñuelas J. Su J. Q. (2018). QMEC: a tool for high-throughput quantitative assessment of microbial functional potential in C, N, P, and S biogeochemical cycling. Sci. China Life Sci. 61, 1451–1462. doi: 10.1007/s11427-018-9364-7 30136056

[ref70] Zhu Y. G. Zhao Y. I. Li B. Huang C. L. Zhang S. Y. Yu S.. (2017). Continental-scale pollution of estuaries with antibiotic resistance genes. Nat. Microbiol. 2, 16270–16277. doi: 10.1038/nmicrobiol.2016.270 28134918

