# Supplementary material for: Changes in the structure and function of rhizosphere soil microbial communities induced by Amaranthus palmeri invasion
Source: Front Microbiol. 2023 Mar 28;14:1114388. doi: 10.3389/fmicb.2023.1114388 (PMC10089265; doi:10.3389/fmicb.2023.1114388)
Supplement: Supplementary file 1 [file Data_Sheet_1.docx]

Supplementary Material

Changes in the structure and function of rhizosphere soil microbial communities induced by *Amaranthus palmeri* invasion

Mei Zhang, Cong Shi, Xueying Li, Kefan Wang, Zhenlu Qiu,

*** Correspondence:** Fuchen Shi: fcshi@nankai.edu.cn

# Supplementary Figures and Tables

## Supplementary Figures

**Supplementary Figure 1.** Location of the study area and sample sites.


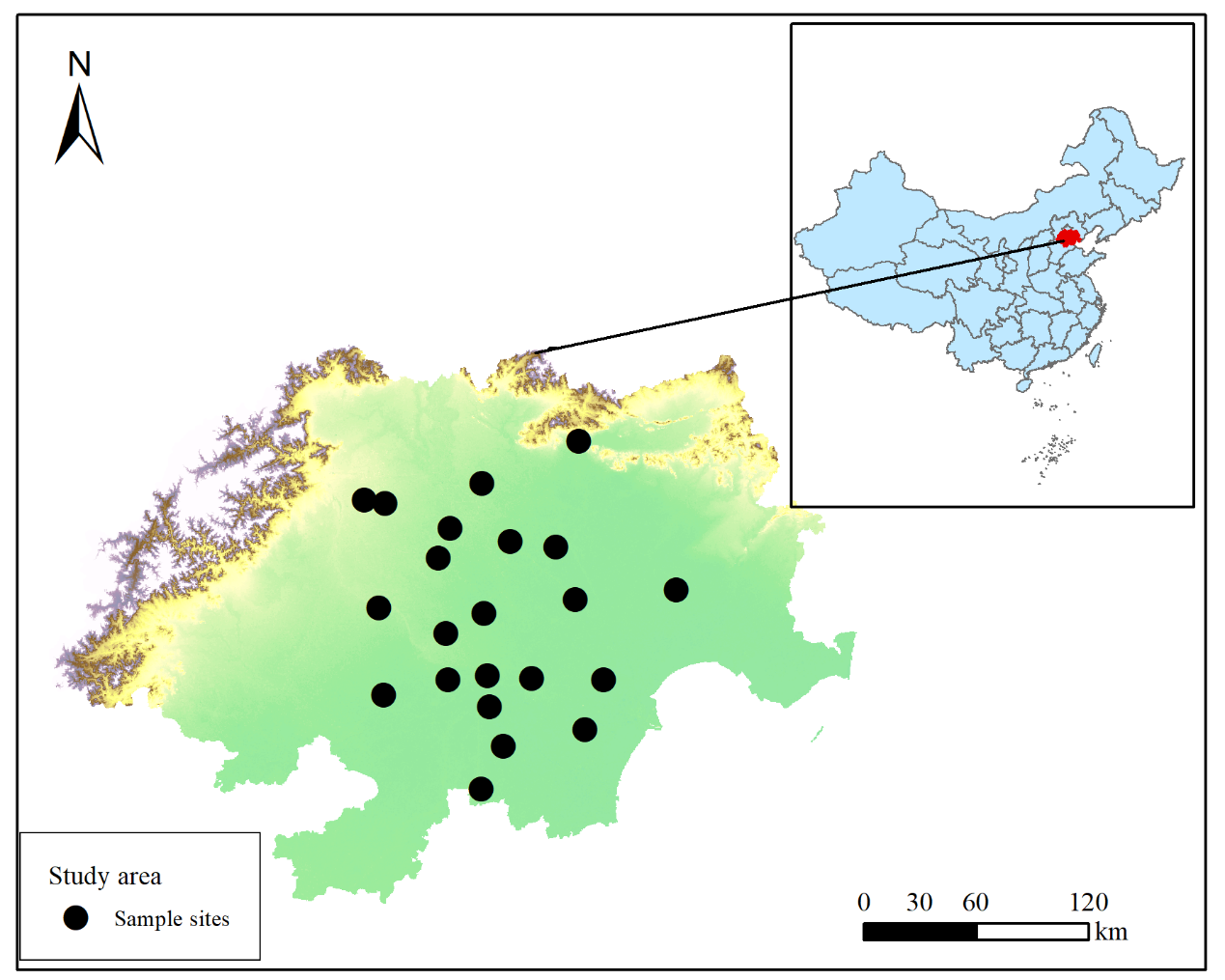


**Supplementary Figure 2.** Dominant taxonomic groups at phylum level in *A*. *palmeri* (AP) and native (N) rhizosphere soils across 22 sites.


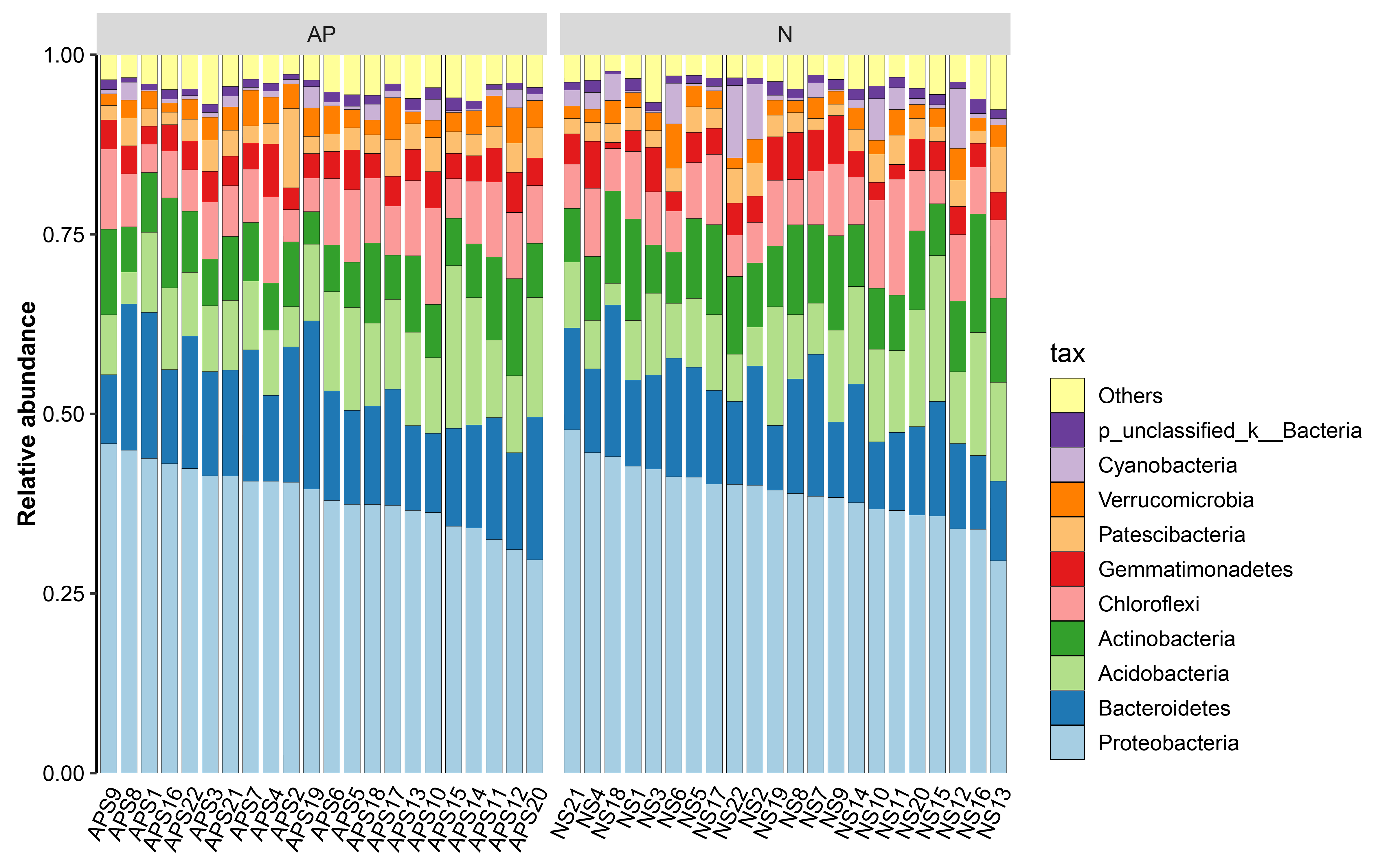


**Supplementary Figure 3.** Dominant taxonomic groups at family level in *A*. *palmeri* (AP) and native (N) rhizosphere soils across 22 sites.


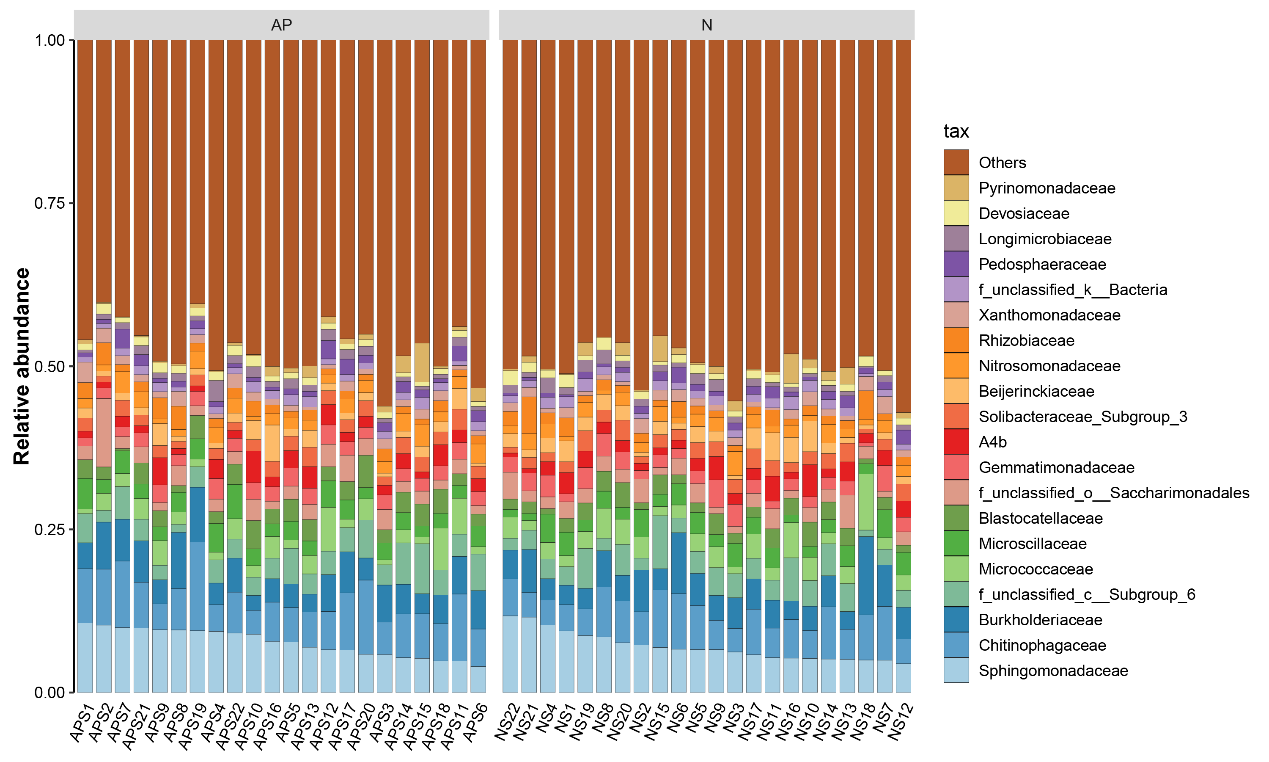


**Supplementary Figure 4.** Comparison the abundance of functional genes in the north and south area in *A*. *palmeri* (A) invaded ecosystems and native (B) ecosystems.


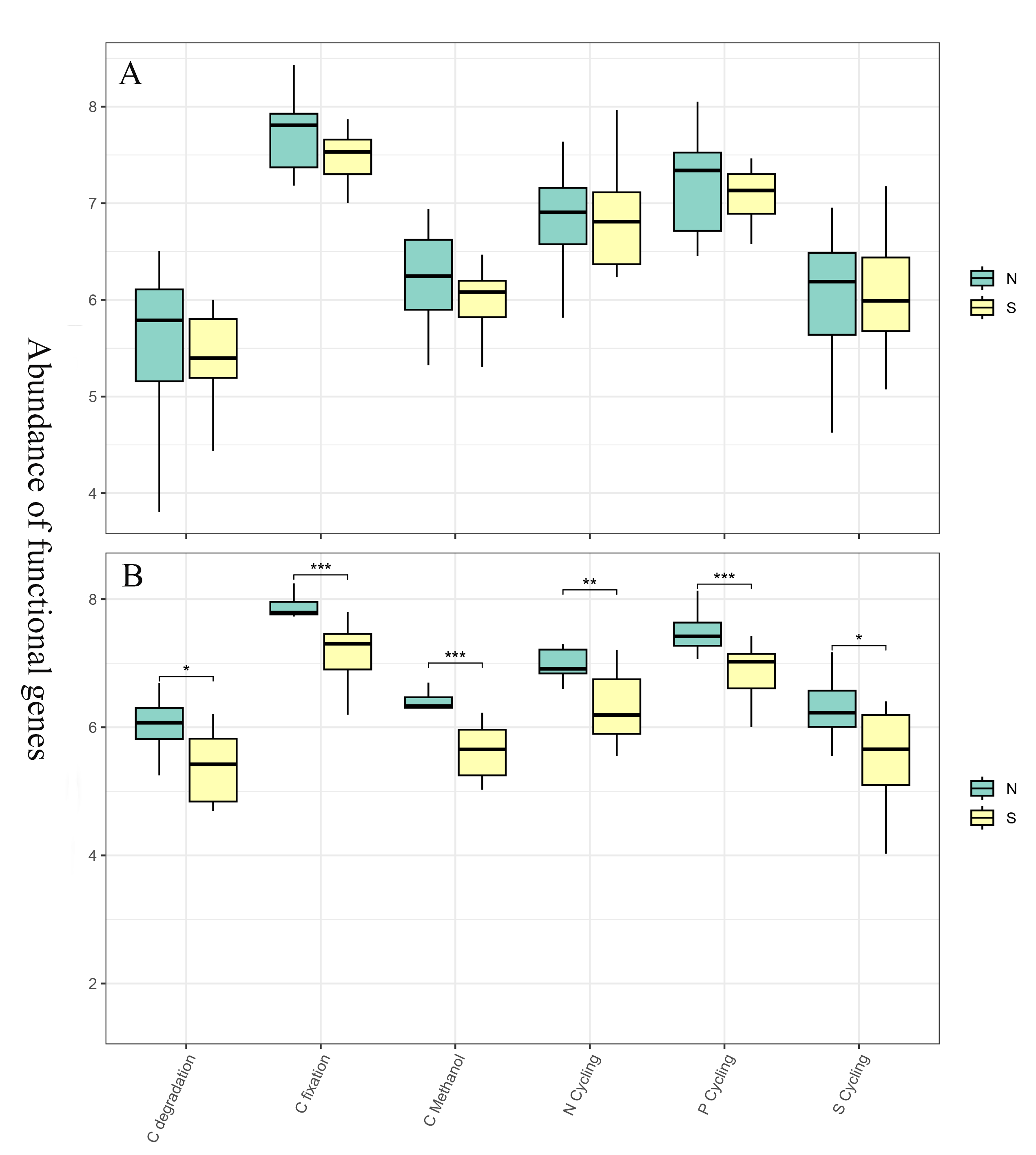


**Supplementary Figure 5.** Ecological relationships between the average standardized abundance of the keystone OTUs and the abundance of functional genes involved in CNPS cycling in *A*. *palmeri* (A) and native (B) rhizosphere soils. Both OTUs and genes were ln transformed.


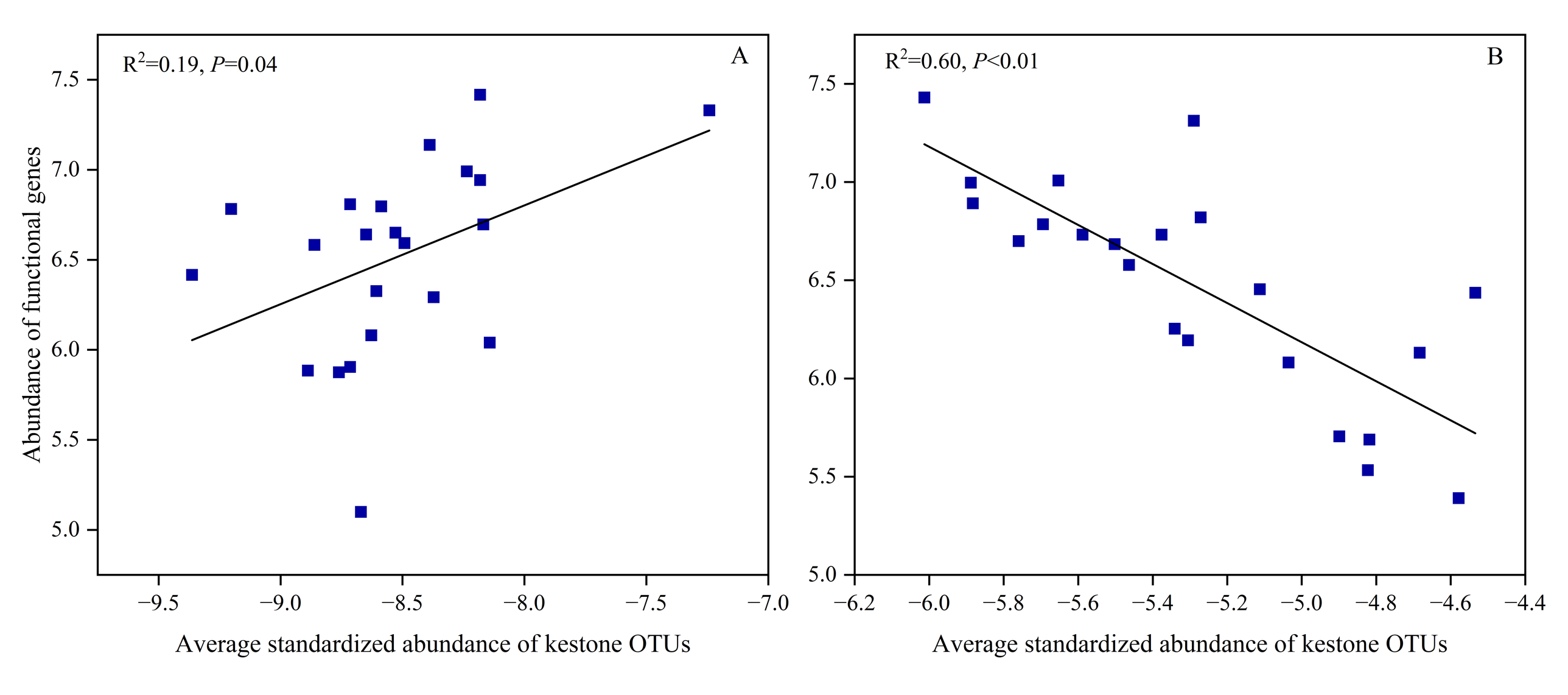


**Supplementary Figure 6.** Heatmap of the correlations between keystone OTUs and edaphic variables in *A*. *palmeri* (a) and native (b) rhizosphere soils. Red circles denote positive correlations, and blue circles denote negative correlations.


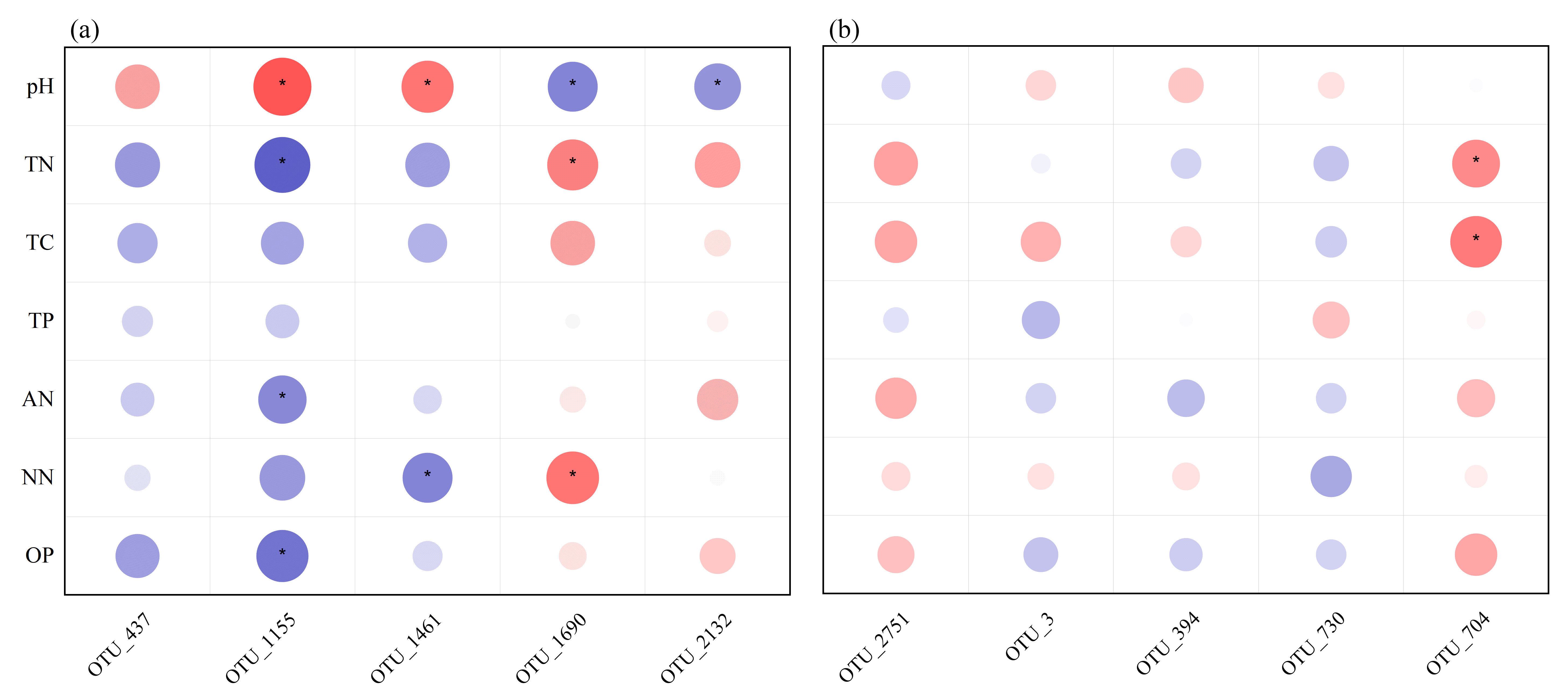


Significant level: * *P* < 0.05.

Abbreviation: TN, total nitrogen; TC, total carbon; TP, total phosphorus; AN, ammonium nitrogen; NN, nitrate nitrogen; AP, available phosphorus.

**Supplementary Figure 7.** Heatmap of the correlations between functional genes involved in CNPS cycling and edaphic variables in *A*. *palmeri* (a) and native (b) rhizosphere soils. Red circles denote positive correlations, and blue circles denote negative correlations.


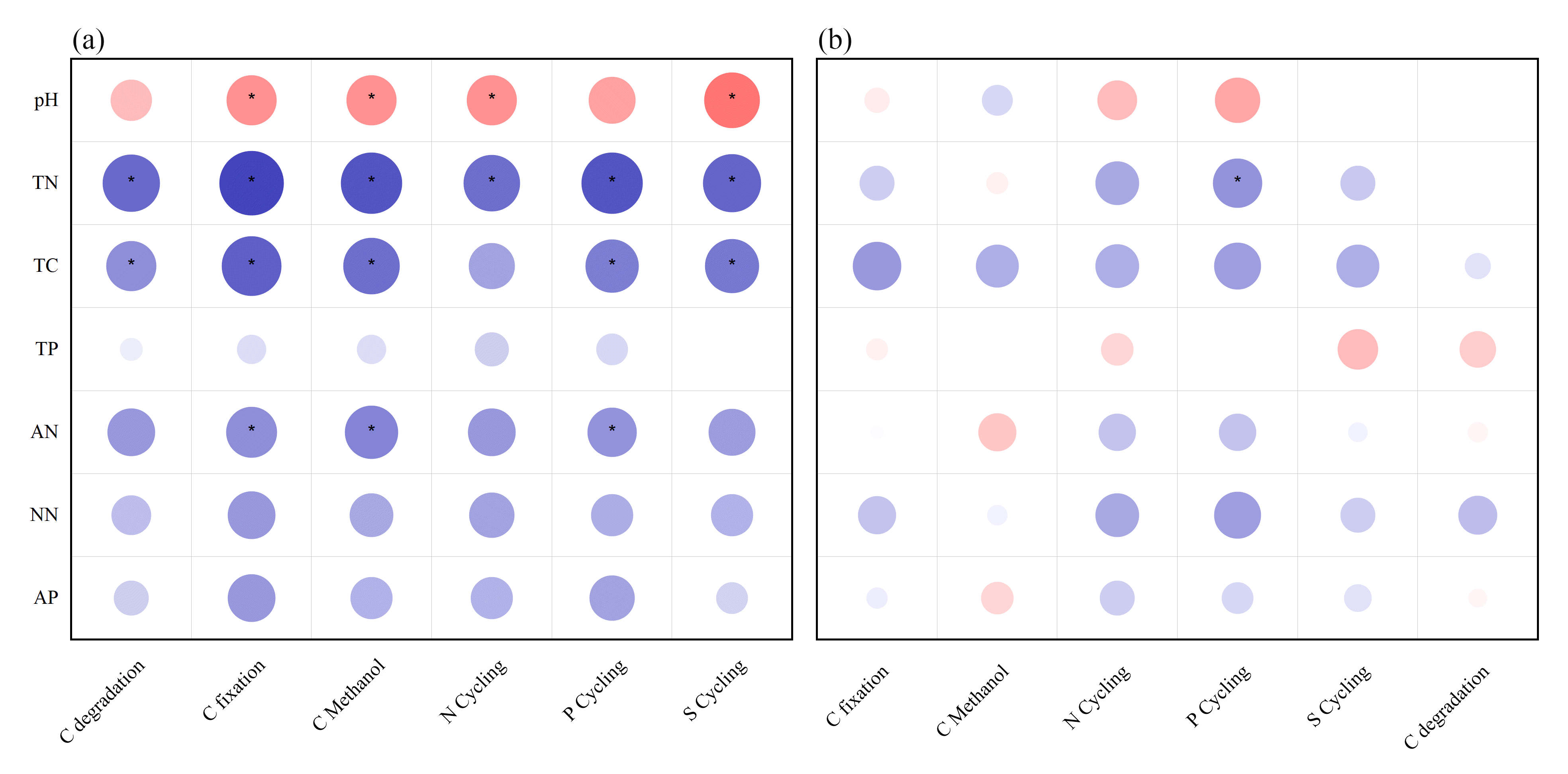


Significant level: * *P* < 0.05.

Abbreviation: TN, total nitrogen; TC, total carbon; TP, total phosphorus; AN, ammonium nitrogen; NN, nitrate nitrogen; AP, available phosphorus.

## 1.2 Supplementary Tables

**Supplementary Table 1.** General characteristics of the 22 sampling sites.

| Site | Province | Longitude | Latitude | Mean annual temperature | Mean annual precipitation | Habitat types | Dominant accompanying species in invasive patches | Dominant species in native patches |
| --- | --- | --- | --- | --- | --- | --- | --- | --- |
| 1 | Tianjin | 116.9238 | 38.73931 | 506 | 12.96 | Wasteland neighbouring roadside | *Chloris virgata,*  *Salsola collina* | *Phragmites australis, Digitaria sanguinalis,Chloris virgata, Artemisia lavandulifolia, Chenopodium glaucum, Salsola collina* |
| 2 | Tianjin | 117.0295 | 38.9025 | 515 | 12.92 | Wasteland neighbouring roadside | *Setaria viridis,*  *Chenopodium album* | *Setaria viridis, Suaeda glauca* |
| 3 | Tianjin | 116.9630 | 39.0493 | 520 | 12.84 | Wasteland | *Amaranthus viridis* | *Setaria viridis, Amaranthus viridis, Abutilon theophrasti, Xanthium sibiricum* |
| 4 | Tianjin | 117.1638 | 39.1558 | 566 | 13.38 | Wasteland | *Setaria viridis* | *Setaria viridis, Digitaria sanguinalis, Amaranthus viridis, Chenopodium album* |
| 5 | Tianjin | 117.2821 | 39.6460 | 549 | 11.95 | Wasteland neighbouring roadside | *Portulaca oleracea* | *Setaria viridis, Artemisia lavandulifolia* |
| 6 | Tianjin | 117.3745 | 39.4490 | 554 | 12.13 | Wasteland | *Portulaca oleracea* | *Setaria viridis, Chenopodium album, Suaeda glauca, Portulaca oleracea* |
| 7 | Tianjin | 117.5103 | 39.1515 | 551 | 12.43 | Wasteland neighbouring roadside | *Setaria viridis* | *Amaranthus viridis, Xanthium strumarium* |
| 8 | Tianjin | 117.4212 | 38.9636 | 555 | 13.03 | Wasteland neighbouring roadside | *Portulaca oleracea* | *Chenopodium album, Setaria viridis* |
| 9 | Tianjin | 116.9363 | 39.3983 | 535 | 12.39 | Wasteland neighbouring roadside | *Setaria viridis* | *Kochia scoparia, Setaria viridis* |
| 10 | Tianjin | 116.9534 | 39.1658 | 532 | 12.69 | Wasteland neighbouring roadside | *Chloris virgata* | *Artemisia scoparia, Kochia scoparia, Setaria viridis, Digitaria sanguinalis,Chloris virgata* |
| 11 | Tianjin | 117.3912 | 40.0390 | 564 | 11.73 | Wasteland neighbouring roadside | *Digitaria sanguinalis* | *Kochia scoparia, Setaria viridis, Digitaria sanguinalis,Chloris virgata* |
| 12 | Tianjin | 117.8583 | 39.4867 | 588 | 11.79 | Wasteland neighbouring roadside | *Eleusine indica* | *Kochia scoparia, Echinochloa crusgalli , Digitaria sanguinalis,Eleusine indica, Chloris virgata* |
| 13 | Hebei | 117.0607 | 39.6670 | 543 | 11.85 | Wasteland neighbouring roadside | *Chenopodium album* | *Abutilon theophrasti, Chenopodium album, Digitaria sanguinalis* |
| 14 | Hebei | 116.9268 | 39.8829 | 538 | 11.68 | Wasteland neighbouring roadside | *Setaria viridis* | *Amaranthus viridis, Setaria viridis, Kochia scoparia* |
| 15 | Hebei | 116.7165 | 39.6035 | 546 | 12.16 | Wasteland | *Eleusine indica* | *Xanthium strumarium, Eleusine indica* |
| 16 | Beijing | 116.7736 | 39.7145 | 545 | 11.92 | Wasteland | *Setaria viridis* | *Xanthium strumarium, Setaria viridis, Eleusine indica* |
| 17 | Beijing | 116.4612 | 39.8071 | 570 | 12.31 | Wasteland | *Kochia scoparia* | *Kochia scoparia, Setaria viridis* |
| 18 | Beijing | 116.3595 | 39.8209 | 571 | 12.40 | Wasteland | *Setaria viridis* | *Chenopodium album, Setaria viridis, Abutilon theophrasti* |
| 19 | Hebei | 116.7545 | 39.3228 | 535 | 12.39 | Wasteland neighbouring roadside | *Eleusine indica* | *Xanthium strumarium, Eleusine indica, Setaria viridis* |
| 20 | Hebei | 116.4299 | 39.4181 | 556 | 12.21 | Wasteland | *Chenopodium album* | *Chenopodium album, Digitaria sanguinalis, Abutilon theophrasti, Setaria viridis* |
| 21 | Hebei | 116.7613 | 39.1514 | 530 | 12.62 | Wasteland neighbouring roadside | *Amaranthus viridis* | *Setaria viridis, Eleusine indica, Digitaria sanguinalis, Amaranthus viridis* |
| 22 | Hebei | 116.4547 | 39.0929 | 512 | 12.67 | Wasteland neighbouring roadside | *Eleusine indica* | *Eleusine indica, Chloris virgata, Echinochloa crusgalli* |

**Supplementary Table 2.** 71 functional genes involved in CNPS cycling determined by QMEC technology.

| **Class** | **Gene** | **Function** |
| --- | --- | --- |
| C degradation | gam | glucoamylase |
| C degradation | abfA | arabinofuranosidase |
| C degradation | xylA | xylose isomerase |
| C degradation | exg | exoglucanase |
| C degradation | PG1 | pectinase |
| C degradation | mnp | manganese peroxidase |
| C degradation | gmGDH | glucose dehydrogenase |
| C degradation | Isop | Isopullulanase |
| C degradation | amyX | AmyX/pullulanase |
| C degradation | apu | Amylopullulanase |
| C degradation | amyA | Alpha amylase |
| C degradation | manA | Mannanase |
| C degradation | CDH | Cellobiose dehydrogenase |
| C degradation | naglu | α-N-acetylglucosaminidase |
| C degradation | chiA | endochitinase |
| C degradation | exc | exochitinase |
| C degradation | glx | Glyoxal oxidase |
| C degradation | lig | Lignin peroxidase |
| C degradation | pox | Phenol oxidase |
| C fixation | rbcL | rubisCO large chain |
| C fixation | aclB | ATP-citrate lyase beta-subunit |
| C fixation | korA | 2-oxoglutarate ferredoxin oxidoreductase |
| C fixation | acsA | carbon-monoxide dehydrogenase |
| C fixation | acsE | 5-methyltetrahydrofolate corrinoid/iron sulfur protein methyltransferase |
| C fixation | acsB | acetyl-CoA synthase |
| C fixation | accA | acetyl-CoA carboxylase carboxyl transferase |
| C fixation | pccA | propionyl-CoA carboxylase alpha |
| C fixation | smtA | succinyl-CoA:(S)-malate CoA transferase |
| C fixation | mct | mesaconyl-CoA C1-C4 CoA transferase |
| C fixation | frdA | fumarate reductase flavoprotein subunit |
| C fixation | mcrA | methyl-coenzyme M reductase alpha subunit |
| C fixation | cdaR | carbohydrate diacid regulon transcriptional regulator |
| Methane metabolism | mmox | methane monooxygenase component A alpha chain |
| Methane metabolism | pmoA | methane/ammonia monooxygenase subunit A |
| Methane metabolism | mxa | methanol dehydrogenase (cytochrome c) subunit |
| Methane metabolism | emGDH | methanol dehydrogenase |
| N Cycling | gdh | glutamate dehydrogenase |
| N Cycling | ureC | urease |
| N Cycling | nifH | nitrogenase iron protein |
| N Cycling | hzo | hydrazine oxidase |
| N Cycling | hzsA | hydrazine synthase subunit A |
| N Cycling | hzsB | hydrazine synthase subunit B |
| N Cycling | amoA-1 | ammonia monooxygenase α-subunit |
| N Cycling | amoA-2 | ammonia monooxygenase α-subunit |
| N Cycling | amoB | ammonia monooxygenase β-subunit |
| N Cycling | hao | hydroxylamine oxidoreductase |
| N Cycling | nxrA | nitrite oxidoreductase |
| N Cycling | narG | nitrate reductase alpha chain |
| N Cycling | napA | periplasmic nitrate reductase |
| N Cycling | nasA | assimilatory nitrate reductase catalytic subunit |
| N Cycling | nirS-1 | nitrite reductase (NO-forming) |
| N Cycling | nirS-2 | nitrite reductase (NO-forming) |
| N Cycling | nirS-3 | nitrite reductase (NO-forming) |
| N Cycling | nirK-1 | nitrite reductase (NO-forming) |
| N Cycling | nirK-2 | nitrite reductase (NO-forming) |
| N Cycling | nirK-3 | nitrite reductase (NO-forming) |
| N Cycling | nosZ-1 | nitrous-oxide reductase |
| N Cycling | nosZ-2 | nitrous-oxide reductase |
| P Cycling | ppk | polyphosphate kinase |
| P Cycling | ppx | exopolyphosphatase |
| P Cycling | phoD | alkaline phosphatase D |
| P Cycling | phoX | alkaline phosphatase/Pho regulon |
| P Cycling | bpp | β-propeller phytase |
| P Cycling | cphy | cystein phytase |
| P Cycling | pqqC | pyrroloquinoline-quinone synthase |
| P Cycling | phnK | putative phosphonate transport system ATP-binding protein |
| S Cycling | dsrA | sulfite reductase alpha subunit |
| S Cycling | dsrB | sulfite reductase beta subunit |
| S Cycling | apsA | adenosine-5'-phosphosulfate reductase subunit A |
| S Cycling | YedZ | sulfite oxidase |
| S Cycling | SoxY | sulfer oxidation protein |

**Supplementary Table 3.** α diversity (richness, Chao1, ACE, Shannon and Simpson indices) in *A*. *palmeri* (AP) and native (N) rhizosphere soils across 22 sampling plots.

| sample | Shannon | Simpson | Richness | Chao1 | Ace |
| --- | --- | --- | --- | --- | --- |
| APS1 | 1.93 | 0.6911 | 8.46 | 8.66 | 8.70 |
| APS2 | 1.88 | 0.6895 | 8.44 | 8.67 | 8.72 |
| APS3 | 2.00 | 0.6921 | 8.70 | 8.89 | 8.94 |
| APS4 | 1.96 | 0.6917 | 8.52 | 8.72 | 8.75 |
| APS5 | 2.00 | 0.6921 | 8.74 | 8.92 | 8.96 |
| APS6 | 2.00 | 0.6922 | 8.72 | 8.91 | 8.94 |
| APS7 | 1.94 | 0.6911 | 8.46 | 8.62 | 8.65 |
| APS8 | 1.90 | 0.6909 | 8.39 | 8.60 | 8.66 |
| APS9 | 1.95 | 0.6904 | 8.62 | 8.82 | 8.86 |
| APS10 | 1.96 | 0.6913 | 8.65 | 8.84 | 8.89 |
| APS11 | 1.90 | 0.6902 | 8.37 | 8.61 | 8.65 |
| APS12 | 1.93 | 0.6898 | 8.56 | 8.79 | 8.83 |
| APS13 | 1.98 | 0.6915 | 8.71 | 8.88 | 8.92 |
| APS14 | 1.99 | 0.6919 | 8.70 | 8.92 | 8.96 |
| APS15 | 1.98 | 0.6917 | 8.68 | 8.88 | 8.92 |
| APS16 | 1.95 | 0.6908 | 8.62 | 8.82 | 8.86 |
| APS17 | 1.97 | 0.6918 | 8.65 | 8.89 | 8.92 |
| APS18 | 1.97 | 0.6905 | 8.70 | 8.93 | 8.98 |
| APS19 | 1.90 | 0.6907 | 8.41 | 8.64 | 8.68 |
| APS20 | 1.96 | 0.6914 | 8.57 | 8.80 | 8.83 |
| APS21 | 1.95 | 0.6913 | 8.57 | 8.77 | 8.82 |
| APS22 | 1.95 | 0.6913 | 8.61 | 8.84 | 8.89 |
| NS1 | 1.96 | 0.6915 | 8.61 | 8.79 | 8.84 |
| NS2 | 1.93 | 0.6909 | 8.54 | 8.77 | 8.82 |
| NS3 | 2.01 | 0.6921 | 8.73 | 8.90 | 8.94 |
| NS4 | 1.96 | 0.6908 | 8.64 | 8.82 | 8.86 |
| NS5 | 1.97 | 0.6917 | 8.61 | 8.79 | 8.82 |
| NS6 | 1.93 | 0.6913 | 8.53 | 8.76 | 8.80 |
| NS7 | 1.95 | 0.6919 | 8.52 | 8.70 | 8.76 |
| NS8 | 1.92 | 0.6906 | 8.50 | 8.70 | 8.75 |
| NS9 | 1.97 | 0.6917 | 8.62 | 8.82 | 8.86 |
| NS10 | 1.95 | 0.6909 | 8.65 | 8.87 | 8.90 |
| NS11 | 1.95 | 0.6910 | 8.58 | 8.78 | 8.83 |
| NS12 | 1.98 | 0.6919 | 8.63 | 8.85 | 8.89 |
| NS13 | 1.99 | 0.6920 | 8.67 | 8.86 | 8.90 |
| NS14 | 2.00 | 0.6920 | 8.74 | 8.93 | 8.98 |
| NS15 | 1.97 | 0.6917 | 8.65 | 8.83 | 8.87 |
| NS16 | 1.95 | 0.6905 | 8.66 | 8.85 | 8.90 |
| NS17 | 1.95 | 0.6912 | 8.54 | 8.74 | 8.79 |
| NS18 | 1.78 | 0.6854 | 8.14 | 8.37 | 8.42 |
| NS19 | 1.98 | 0.6913 | 8.70 | 8.85 | 8.90 |
| NS20 | 1.97 | 0.6910 | 8.64 | 8.79 | 8.83 |
| NS21 | 1.92 | 0.6899 | 8.51 | 8.71 | 8.74 |
| NS22 | 1.92 | 0.6903 | 8.55 | 8.75 | 8.79 |
| AP vs N | ns | ns | ns | ns | ns |

**Supplementary Table 4.** Kruskal-Wallis test at the phylum level.

| tax | p | p.adj | p.format | p.signif | method |
| --- | --- | --- | --- | --- | --- |
| Acidobacteria | 0.541671 | 1 | 0.542 | ns | Kruskal-Wallis |
| **Actinobacteria** | **0.016656** | **0.48** | **0.017** | ***** | **Kruskal-Wallis** |
| Armatimonadetes | 0.213481 | 1 | 0.213 | ns | Kruskal-Wallis |
| **Bacteroidetes** | **0.046024** | **1** | **0.046** | ***** | **Kruskal-Wallis** |
| BRC1 | 0.105316 | 1 | 0.105 | ns | Kruskal-Wallis |
| Chlamydiae | 0.078332 | 1 | 0.078 | ns | Kruskal-Wallis |
| Chloroflexi | 0.925196 | 1 | 0.925 | ns | Kruskal-Wallis |
| Cyanobacteria | 0.133033 | 1 | 0.133 | ns | Kruskal-Wallis |
| Deinococcus-Thermus | 0.452578 | 1 | 0.453 | ns | Kruskal-Wallis |
| Dependentiae | 0.269934 | 1 | 0.27 | ns | Kruskal-Wallis |
| Elusimicrobia | 0.459656 | 1 | 0.46 | ns | Kruskal-Wallis |
| Entotheonellaeota | 0.672611 | 1 | 0.673 | ns | Kruskal-Wallis |
| FBP | 0.452578 | 1 | 0.453 | ns | Kruskal-Wallis |
| FCPU426 | 0.653712 | 1 | 0.654 | ns | Kruskal-Wallis |
| Fibrobacteres | 0.065368 | 1 | 0.065 | ns | Kruskal-Wallis |
| Firmicutes | 0.906572 | 1 | 0.907 | ns | Kruskal-Wallis |
| Gemmatimonadetes | 0.888 | 1 | 0.888 | ns | Kruskal-Wallis |
| Hydrogenedentes | 0.208632 | 1 | 0.209 | ns | Kruskal-Wallis |
| Latescibacteria | 0.318343 | 1 | 0.318 | ns | Kruskal-Wallis |
| **Nitrospirae** | **0.018912** | **0.53** | **0.019** | ***** | **Kruskal-Wallis** |
| Omnitrophicaeota | 0.073998 | 1 | 0.074 | ns | Kruskal-Wallis |
| Patescibacteria | 0.411338 | 1 | 0.411 | ns | Kruskal-Wallis |
| Planctomycetes | 0.689868 | 1 | 0.69 | ns | Kruskal-Wallis |
| Proteobacteria | 0.925196 | 1 | 0.925 | ns | Kruskal-Wallis |
| Rokubacteria | 0.452578 | 1 | 0.453 | ns | Kruskal-Wallis |
| Spirochaetes | 0.191568 | 1 | 0.192 | ns | Kruskal-Wallis |
| Verrucomicrobia | 0.082392 | 1 | 0.082 | ns | Kruskal-Wallis |

**Supplementary Table 5.** Kruskal-Wallis test at the family level.

| tax | p | p.format | p.signif | method |
| --- | --- | --- | --- | --- |
| 0319-6G20 | 0.481321 | 0.4813 | ns | Kruskal-Wallis |
| 27F-1492R | 0.547987 | 0.548 | ns | Kruskal-Wallis |
| 37-13 | 0.347763 | 0.3478 | ns | Kruskal-Wallis |
| 67-14 | 0.078332 | 0.0783 | ns | Kruskal-Wallis |
| A21b | 0.364631 | 0.3646 | ns | Kruskal-Wallis |
| A4b | 0.814421 | 0.8144 | ns | Kruskal-Wallis |
| AB1 | 0.070602 | 0.0706 | ns | Kruskal-Wallis |
| Acetobacteraceae | 0.05426 | 0.0543 | ns | Kruskal-Wallis |
| Acidiferrobacteraceae | 0.630153 | 0.6302 | ns | Kruskal-Wallis |
| Aeromonadaceae | 0.403426 | 0.4034 | ns | Kruskal-Wallis |
| AKIW781 | 0.269934 | 0.2699 | ns | Kruskal-Wallis |
| AKYG1722 | 0.496057 | 0.4961 | ns | Kruskal-Wallis |
| **AKYH767** | **0.022795** | **0.0228** | ***** | **Kruskal-Wallis** |
| Alicyclobacillaceae | 0.990628 | 0.9906 | ns | Kruskal-Wallis |
| Anaerolineaceae | 0.33586 | 0.3359 | ns | Kruskal-Wallis |
| **Archangiaceae** | **0.012843** | **0.0128** | ***** | **Kruskal-Wallis** |
| **Ardenticatenaceae** | **0.03464** | **0.0346** | ***** | **Kruskal-Wallis** |
| **Azospirillaceae** | **0.03464** | **0.0346** | ***** | **Kruskal-Wallis** |
| B1-7BS | 0.962554 | 0.9626 | ns | Kruskal-Wallis |
| Bacillaceae | 0.411338 | 0.4113 | ns | Kruskal-Wallis |
| bacteriap25 | 0.557314 | 0.5573 | ns | Kruskal-Wallis |
| Bacteriovoracaceae | 0.307192 | 0.3072 | ns | Kruskal-Wallis |
| Bdellovibrionaceae | 0.778195 | 0.7782 | ns | Kruskal-Wallis |
| Beijerinckiaceae | 0.173383 | 0.1734 | ns | Kruskal-Wallis |
| BIrii41 | 0.796253 | 0.7963 | ns | Kruskal-Wallis |
| Blastocatellaceae | 0.05426 | 0.0543 | ns | Kruskal-Wallis |
| Blfdi19 | 0.082392 | 0.0824 | ns | Kruskal-Wallis |
| Bogoriellaceae | 0.274148 | 0.2741 | ns | Kruskal-Wallis |
| Burkholderiaceae | 0.655612 | 0.6556 | ns | Kruskal-Wallis |
| C2U | 1 | 1 | ns | Kruskal-Wallis |
| Caldilineaceae | 0.888 | 0.888 | ns | Kruskal-Wallis |
| **Caulobacteraceae** | **0.048644** | **0.0486** | ***** | **Kruskal-Wallis** |
| Cellulomonadaceae | 0.391566 | 0.3916 | ns | Kruskal-Wallis |
| Cellvibrionaceae | 0.557314 | 0.5573 | ns | Kruskal-Wallis |
| Chitinophagaceae | 0.095603 | 0.0956 | ns | Kruskal-Wallis |
| Chloroflexaceae | 0.121335 | 0.1213 | ns | Kruskal-Wallis |
| Chromobacteriaceae | 0.840267 | 0.8403 | ns | Kruskal-Wallis |
| Chthoniobacteraceae | 0.231266 | 0.2313 | ns | Kruskal-Wallis |
| Chthonomonadaceae | 0.698212 | 0.6982 | ns | Kruskal-Wallis |
| Clostridiaceae_1 | 0.887921 | 0.8879 | ns | Kruskal-Wallis |
| Coleofasciculaceae | 0.254894 | 0.2549 | ns | Kruskal-Wallis |
| Coxiellaceae | 0.18844 | 0.1884 | ns | Kruskal-Wallis |
| Crocinitomicaceae | 0.549105 | 0.5491 | ns | Kruskal-Wallis |
| Cryptosporangiaceae | 0.564165 | 0.5642 | ns | Kruskal-Wallis |
| cvE6 | 0.148746 | 0.1487 | ns | Kruskal-Wallis |
| Cyclobacteriaceae | 0.557244 | 0.5572 | ns | Kruskal-Wallis |
| Cytophagaceae | 0.245261 | 0.2453 | ns | Kruskal-Wallis |
| D05-2 | 0.724771 | 0.7248 | ns | Kruskal-Wallis |
| **Deinococcaceae** | **0.018841** | **0.0188** | ***** | **Kruskal-Wallis** |
| Desulfarculaceae | 0.474013 | 0.474 | ns | Kruskal-Wallis |
| Devosiaceae | 0.814421 | 0.8144 | ns | Kruskal-Wallis |
| Diplorickettsiaceae | 0.526237 | 0.5262 | ns | Kruskal-Wallis |
| Dongiaceae | 0.638746 | 0.6387 | ns | Kruskal-Wallis |
| Enterobacteriaceae | 0.1392 | 0.1392 | ns | Kruskal-Wallis |
| Entotheonellaceae | 0.672611 | 0.6726 | ns | Kruskal-Wallis |
| env | 0.943861 | 0.9439 | ns | Kruskal-Wallis |
| Euzebyaceae | 0.347712 | 0.3477 | ns | Kruskal-Wallis |
| Family_XII | 0.533449 | 0.5334 | ns | Kruskal-Wallis |
| Fibrobacteraceae | 0.074427 | 0.0744 | ns | Kruskal-Wallis |
| **Fimbriimonadaceae** | **0.006027** | **0.006** | ****** | **Kruskal-Wallis** |
| Flavobacteriaceae | 0.074437 | 0.0744 | ns | Kruskal-Wallis |
| Gaiellaceae | 0.655612 | 0.6556 | ns | Kruskal-Wallis |
| Geminicoccaceae | 0.133033 | 0.133 | ns | Kruskal-Wallis |
| Gemmataceae | 0.915873 | 0.9159 | ns | Kruskal-Wallis |
| Gemmatimonadaceae | 0.962557 | 0.9626 | ns | Kruskal-Wallis |
| **Geodermatophilaceae** | **0.022795** | **0.0228** | ***** | **Kruskal-Wallis** |
| Glycomycetaceae | 0.767678 | 0.7677 | ns | Kruskal-Wallis |
| Haliangiaceae | 0.672655 | 0.6727 | ns | Kruskal-Wallis |
| Herpetosiphonaceae | 0.110458 | 0.1105 | ns | Kruskal-Wallis |
| **Holosporaceae** | **0.009792** | **0.0098** | ****** | **Kruskal-Wallis** |
| Hydrogenedensaceae | 0.208632 | 0.2086 | ns | Kruskal-Wallis |
| Hymenobacteraceae | 0.541671 | 0.5417 | ns | Kruskal-Wallis |
| Hyphomicrobiaceae | 0.269934 | 0.2699 | ns | Kruskal-Wallis |
| Hyphomonadaceae | 0.925196 | 0.9252 | ns | Kruskal-Wallis |
| Iamiaceae | 0.385128 | 0.3851 | ns | Kruskal-Wallis |
| Ilumatobacteraceae | 0.05426 | 0.0543 | ns | Kruskal-Wallis |
| Intrasporangiaceae | 0.173383 | 0.1734 | ns | Kruskal-Wallis |
| Isosphaeraceae | 0.897207 | 0.8972 | ns | Kruskal-Wallis |
| JG30-KF-CM45 | 0.742445 | 0.7424 | ns | Kruskal-Wallis |
| **KD3-10** | **0.038441** | **0.0384** | ***** | **Kruskal-Wallis** |
| KD3-93 | 0.786799 | 0.7868 | ns | Kruskal-Wallis |
| KF-JG30-B3 | 0.93446 | 0.9345 | ns | Kruskal-Wallis |
| Kiloniellaceae | 0.395644 | 0.3956 | ns | Kruskal-Wallis |
| Kineosporiaceae | 0.22223 | 0.2222 | ns | Kruskal-Wallis |
| Labraceae | 0.230459 | 0.2305 | ns | Kruskal-Wallis |
| Legionellaceae | 0.81437 | 0.8144 | ns | Kruskal-Wallis |
| Longimicrobiaceae | 0.943861 | 0.9439 | ns | Kruskal-Wallis |
| Magnetospiraceae | 0.776572 | 0.7766 | ns | Kruskal-Wallis |
| Methyloligellaceae | 0.301683 | 0.3017 | ns | Kruskal-Wallis |
| Methylomirabilaceae | 0.749933 | 0.7499 | ns | Kruskal-Wallis |
| Methylophilaceae | 0.082392 | 0.0824 | ns | Kruskal-Wallis |
| Microbacteriaceae | 0.057265 | 0.0573 | ns | Kruskal-Wallis |
| Micrococcaceae | 0.280258 | 0.2803 | ns | Kruskal-Wallis |
| Micromonosporaceae | 0.110458 | 0.1105 | ns | Kruskal-Wallis |
| Micropepsaceae | 0.823494 | 0.8235 | ns | Kruskal-Wallis |
| Microscillaceae | 0.42483 | 0.4248 | ns | Kruskal-Wallis |
| Microtrichaceae | 0.290642 | 0.2906 | ns | Kruskal-Wallis |
| Midichloriaceae | 0.391132 | 0.3911 | ns | Kruskal-Wallis |
| mle1-27 | 0.851048 | 0.851 | ns | Kruskal-Wallis |
| Moraxellaceae | 0.943861 | 0.9439 | ns | Kruskal-Wallis |
| Mycobacteriaceae | 0.280258 | 0.2803 | ns | Kruskal-Wallis |
| Myxococcaceae | 0.347576 | 0.3476 | ns | Kruskal-Wallis |
| Nakamurellaceae | 0.067085 | 0.0671 | ns | Kruskal-Wallis |
| Nannocystaceae | 0.638746 | 0.6387 | ns | Kruskal-Wallis |
| Nitrosomonadaceae | 0.466827 | 0.4668 | ns | Kruskal-Wallis |
| **Nitrospiraceae** | **0.025754** | **0.0258** | ***** | **Kruskal-Wallis** |
| Nocardiaceae | 0.08662 | 0.0866 | ns | Kruskal-Wallis |
| Nocardioidaceae | 0.133033 | 0.133 | ns | Kruskal-Wallis |
| Nostocaceae | 0.188393 | 0.1884 | ns | Kruskal-Wallis |
| NS11-12_marine_group | 0.307158 | 0.3072 | ns | Kruskal-Wallis |
| NS9_marine_group | 0.814415 | 0.8144 | ns | Kruskal-Wallis |
| Oligoflexaceae | 0.180916 | 0.1809 | ns | Kruskal-Wallis |
| Opitutaceae | 0.638746 | 0.6387 | ns | Kruskal-Wallis |
| P3OB-42 | 0.638746 | 0.6387 | ns | Kruskal-Wallis |
| Paenibacillaceae | 1 | 1 | ns | Kruskal-Wallis |
| Paracaedibacteraceae | 0.280207 | 0.2802 | ns | Kruskal-Wallis |
| Parachlamydiaceae | 0.269867 | 0.2699 | ns | Kruskal-Wallis |
| Pedosphaeraceae | 0.42483 | 0.4248 | ns | Kruskal-Wallis |
| Peptococcaceae | 0.636816 | 0.6368 | ns | Kruskal-Wallis |
| Peptostreptococcaceae | 0.786792 | 0.7868 | ns | Kruskal-Wallis |
| Phaselicystidaceae | 0.851048 | 0.851 | ns | Kruskal-Wallis |
| Phormidiaceae | 0.093105 | 0.0931 | ns | Kruskal-Wallis |
| Phycisphaeraceae | 0.398103 | 0.3981 | ns | Kruskal-Wallis |
| Planococcaceae | 0.778188 | 0.7782 | ns | Kruskal-Wallis |
| Pleomorphomonadaceae | 0.934483 | 0.9345 | ns | Kruskal-Wallis |
| Polyangiaceae | 0.280258 | 0.2803 | ns | Kruskal-Wallis |
| Promicromonosporaceae | 0.925141 | 0.9251 | ns | Kruskal-Wallis |
| Propionibacteriaceae | 0.296137 | 0.2961 | ns | Kruskal-Wallis |
| Pseudohongiellaceae | 0.897113 | 0.8971 | ns | Kruskal-Wallis |
| Pseudomonadaceae | 0.496057 | 0.4961 | ns | Kruskal-Wallis |
| Pseudonocardiaceae | 0.481321 | 0.4813 | ns | Kruskal-Wallis |
| Puniceispirillales_Incertae_Sedis | 0.353194 | 0.3532 | ns | Kruskal-Wallis |
| Pyrinomonadaceae | 0.796253 | 0.7963 | ns | Kruskal-Wallis |
| Reyranellaceae | 0.622065 | 0.6221 | ns | Kruskal-Wallis |
| Rhizobiaceae | 0.943861 | 0.9439 | ns | Kruskal-Wallis |
| Rhizobiales_Incertae_Sedis | 0.213481 | 0.2135 | ns | Kruskal-Wallis |
| **Rhodanobacteraceae** | **0.01202** | **0.012** | ***** | **Kruskal-Wallis** |
| Rhodobacteraceae | 0.814421 | 0.8144 | ns | Kruskal-Wallis |
| Rhodocyclaceae | 0.082392 | 0.0824 | ns | Kruskal-Wallis |
| Rhodomicrobiaceae | 0.231036 | 0.231 | ns | Kruskal-Wallis |
| Rhodopirillaceae | 0.869356 | 0.8694 | ns | Kruskal-Wallis |
| Rhodospirillaceae | 0.715969 | 0.716 | ns | Kruskal-Wallis |
| Rhodothermaceae | 0.385044 | 0.385 | ns | Kruskal-Wallis |
| Rickettsiaceae | 0.341616 | 0.3416 | ns | Kruskal-Wallis |
| Roseiflexaceae | 0.42483 | 0.4248 | ns | Kruskal-Wallis |
| **Rubritaleaceae** | **0.025754** | **0.0258** | ***** | **Kruskal-Wallis** |
| Rubrobacteriaceae | 0.057265 | 0.0573 | ns | Kruskal-Wallis |
| Ruminococcaceae | 0.403675 | 0.4037 | ns | Kruskal-Wallis |
| Saccharimonadaceae | 0.076343 | 0.0763 | ns | Kruskal-Wallis |
| Sandaracinaceae | 0.290846 | 0.2908 | ns | Kruskal-Wallis |
| Saprospiraceae | 0.888 | 0.888 | ns | Kruskal-Wallis |
| SC-I-84 | 0.347779 | 0.3478 | ns | Kruskal-Wallis |
| Simkaniaceae | 0.458943 | 0.4589 | ns | Kruskal-Wallis |
| SM2D12 | 0.398103 | 0.3981 | ns | Kruskal-Wallis |
| Sneathiellaceae | 0.136007 | 0.136 | ns | Kruskal-Wallis |
| Solibacteraceae_Subgroup_3 | 0.832689 | 0.8327 | ns | Kruskal-Wallis |
| Solimonadaceae | 0.51859 | 0.5186 | ns | Kruskal-Wallis |
| **Solirubrobacteraceae** | **0.003888** | **0.0039** | ****** | **Kruskal-Wallis** |
| Sphingobacteriaceae | 0.372414 | 0.3724 | ns | Kruskal-Wallis |
| Sphingomonadaceae | 0.290846 | 0.2908 | ns | Kruskal-Wallis |
| Spirosomaceae | 0.180916 | 0.1809 | ns | Kruskal-Wallis |
| Spongiibacteraceae | 0.813248 | 0.8132 | ns | Kruskal-Wallis |
| Sporichthyaceae | 0.169676 | 0.1697 | ns | Kruskal-Wallis |
| SS1-B-06-26 | 0.813477 | 0.8135 | ns | Kruskal-Wallis |
| Stappiaceae | 0.776587 | 0.7766 | ns | Kruskal-Wallis |
| Steroidobacteraceae | 0.31282 | 0.3128 | ns | Kruskal-Wallis |
| Streptomycetaceae | 0.742445 | 0.7424 | ns | Kruskal-Wallis |
| Streptosporangiaceae | 0.166088 | 0.1661 | ns | Kruskal-Wallis |
| Syntrophaceae | 0.569907 | 0.5699 | ns | Kruskal-Wallis |
| Tepidisphaeraceae | 0.841698 | 0.8417 | ns | Kruskal-Wallis |
| Thermoactinomycetaceae | 0.251694 | 0.2517 | ns | Kruskal-Wallis |
| Thermoanaerobaculaceae | 0.42483 | 0.4248 | ns | Kruskal-Wallis |
| Thermomicrobiaceae | 0.417609 | 0.4176 | ns | Kruskal-Wallis |
| Thermomonosporaceae | 0.359711 | 0.3597 | ns | Kruskal-Wallis |
| Thermosynechococcaceae | 0.715576 | 0.7156 | ns | Kruskal-Wallis |
| TRA3-20 | 0.655612 | 0.6556 | ns | Kruskal-Wallis |
| Trueperaceae | 0.231184 | 0.2312 | ns | Kruskal-Wallis |
| Vermiphilaceae | 0.217816 | 0.2178 | ns | Kruskal-Wallis |
| Verrucomicrobiaceae | 0.121335 | 0.1213 | ns | Kruskal-Wallis |
| VHS-B3-70 | 0.778028 | 0.778 | ns | Kruskal-Wallis |
| Vulgatibacteraceae | 0.241258 | 0.2413 | ns | Kruskal-Wallis |
| WD2101_soil_group | 0.269934 | 0.2699 | ns | Kruskal-Wallis |
| Weeksellaceae | 0.814421 | 0.8144 | ns | Kruskal-Wallis |
| Woeseiaceae | 0.742121 | 0.7421 | ns | Kruskal-Wallis |
| Xanthobacteraceae | 0.796253 | 0.7963 | ns | Kruskal-Wallis |
| Xanthomonadaceae | 0.906572 | 0.9066 | ns | Kruskal-Wallis |
| Xiphinematobacteraceae | 0.457412 | 0.4574 | ns | Kruskal-Wallis |

**Supplementary Table 6.** 52 functional genes measured in the present study.

| Class | Gene_name |
| --- | --- |
| C degradation | abfA |
| C degradation | lig |
| C degradation | cex |
| C degradation | pgu |
| C degradation | glx |
| C degradation | manB |
| C degradation | mnp |
| C degradation | sga |
| C degradation | chiA |
| C degradation | gcd |
| C degradation | xylA |
| C degradation | apu |
| C degradation | iso-plu |
| C fixation | aclB |
| C fixation | mct |
| C fixation | rbcL |
| C fixation | frdA |
| C fixation | korA |
| C fixation | accA |
| C fixation | acsA |
| C fixation | acsE |
| C fixation | pccA |
| C fixation | smtA |
| Methane metabolism | mmoX |
| Methane metabolism | mxaF |
| Methane metabolism | pmoA |
| Methane metabolism | pqq-mdh |
| N Cycling | amoA1 |
| N Cycling | nirS2 |
| N Cycling | nosZ1 |
| N Cycling | ureC |
| N Cycling | narG |
| N Cycling | nifH |
| N Cycling | nirK2 |
| N Cycling | nirS1 |
| N Cycling | amoA2 |
| N Cycling | napA |
| N Cycling | nirS3 |
| N Cycling | nosZ2 |
| N Cycling | amoB |
| N Cycling | nirK1 |
| N Cycling | nirK3 |
| N Cycling | gdhA |
| P Cycling | bpp |
| P Cycling | phoX |
| P Cycling | pqqC |
| P Cycling | phnK |
| P Cycling | phoD |
| P Cycling | ppx |
| S Cycling | apsA |
| S Cycling | soxY |
| S Cycling | dsrA |
| S Cycling | yedZ |

**Supplementary Table 7.** Major topological properties of phylogenetic molecular ecological networks of functional genes in *A*. *palmeri* (AP) and native(N) rhizosphere soils.

| network | AP | N |
| --- | --- | --- |
| node number | 42 | 44 |
| edge number | 154 | 75 |
| net average degree | 7.33 | 3.41 |
| net positive edges | 154 | 75 |
| net negative edges | NA | NA |
| edge density | 0.18 | 0.08 |
| net diameter | 5 | 6 |
| net transitivity | 0.50 | 0.44 |
| clustering coefficient | 0.50 | 0.44 |
| net mean distance | 2.29 | 2.96 |
| Assortativity | 0.07 | 0.23 |
| net degree centrality | 0.24 | 0.15 |
| average neighbors | 9.27 | 4.60 |
| net modularity | 0.39 | 0.57 |
| HarMonicGeoDis | 2.78 | 6.39 |
| mean(clustering coefficient rand) | 0.24±0.03 | 0.12±0.04 |
| mean (HarMonicGeoDis rand) | 2.65±0.07 | 5.07±0.34 |

**Supplementary Table 8.** Correlations between the five keystone taxa and functional genes in *A*. *palmeri* (AP) rhizosphere soils.

|  | otu437 | otu1155 | otu1461 | otu1690 | otu2132 |
| --- | --- | --- | --- | --- | --- |
| abfA | .507* | 0.031 | 0.336 | -0.367 | -0.233 |
| lig | .443* | .469* | 0.195 | -0.295 | -0.129 |
| cex | 0.262 | -0.018 | 0.176 | -0.183 | -0.276 |
| pgu | 0.253 | 0.234 | 0.066 | -0.113 | -0.005 |
| amyA | 0.364 | 0.396 | 0.295 | -0.226 | -.483* |
| cdh | .549** | .615** | 0.4 | -.491* | -.656** |
| glx | .722** | .533* | 0.373 | -0.322 | -.499* |
| manB | .600** | 0.348 | 0.158 | -0.228 | -0.266 |
| mnp | .693** | .632** | 0.336 | -0.419 | -.471* |
| sga | .619** | 0.302 | .565** | -.570** | -.433* |
| chiA | .702** | 0.298 | .519* | -.558** | -.524* |
| gcd | .596** | .525* | 0.213 | -0.311 | -.493* |
| xylA | .577** | 0.338 | .447* | -.457* | -0.258 |
| apu | .432* | 0.103 | 0.202 | -0.192 | -0.258 |
| iso-plu | .662** | .750** | .510* | -.498* | -.681** |
| aclB | .639** | .662** | .464* | -.544** | -.423* |
| mct | .606** | 0.36 | 0.405 | -.432* | -0.334 |
| rbcL | 0.399 | .465* | .665** | -.604** | -.658** |
| frdA | 0.419 | 0.418 | .637** | -.638** | -.593** |
| korA | .722** | .787** | .548** | -.519* | -.641** |
| accA | .757** | 0.39 | .494* | -.518* | -.596** |
| acsA | .692** | .456* | .626** | -.596** | -.546** |
| acsE | .726** | .638** | .483* | -.541** | -.555** |
| pccA | .473* | .439* | .496* | -.425* | -0.414 |
| smtA | .541** | 0.226 | 0.377 | -0.416 | -0.171 |
| mmoX | .628** | .622** | 0.358 | -0.322 | -.478* |
| mxaF | .626** | 0.41 | .441* | -0.406 | -0.404 |
| pmoA | .626** | .670** | 0.328 | -0.297 | -0.323 |
| pqq-mdh | .552** | .661** | .832** | -.707** | -.698** |
| amoA1 | 0.34 | 0.259 | 0.289 | -0.349 | -0.215 |
| nirS2 | 0.303 | 0.374 | .626** | -.637** | -.518* |
| nosZ1 | .607** | .582** | .789** | -.763** | -.719** |
| ureC | 0.285 | 0.331 | .489* | -.438* | -.441* |
| narG | .433* | 0.4 | .591** | -.768** | -.658** |
| nifH | 0.377 | .606** | .716** | -.595** | -.534* |
| nirK2 | .668** | .500* | .717** | -.721** | -.713** |
| nirS1 | 0.193 | 0.337 | .611** | -.637** | -.493* |
| amoA2 | 0.28 | 0.401 | -0.074 | 0.012 | -0.157 |
| napA | .632** | 0.405 | 0.366 | -0.371 | -.534* |
| nirS3 | .588** | 0.225 | 0.237 | -0.278 | -0.163 |
| nosZ2 | .609** | .532* | .753** | -.752** | -.741** |
| amoB | 0.346 | 0.199 | 0.211 | -0.266 | -0.042 |
| nirK1 | .540** | 0.289 | .567** | -.604** | -.586** |
| nirK3 | .663** | .618** | .789** | -.768** | -.714** |
| gdhA | .756** | .473* | .670** | -.837** | -.691** |
| bpp | .652** | .614** | 0.365 | -.460* | -.551** |
| phoX | .656** | 0.399 | 0.346 | -0.338 | -.445* |
| pqqC | 0.304 | .533* | .645** | -.552** | -.544** |
| phnK | .575** | .634** | .645** | -.580** | -.607** |
| phoD | 0.342 | 0.387 | 0.358 | -0.289 | -0.192 |
| ppx | .732** | .655** | .440* | -.454* | -.610** |
| apsA | .713** | .562** | 0.406 | -.502* | -.456* |
| soxY | .735** | .719** | .534* | -.564** | -.597** |
| dsrA | .601** | .451* | .595** | -.685** | -.494* |
| yedZ | .649** | .482* | .524* | -.539** | -0.318 |

**Supplementary Table 9.** Correlations between the five keystone taxa and functional genes in native (N) rhizosphere soils.

|  | otu2751 | otu3 | otu394 | otu730 | otu704 |
| --- | --- | --- | --- | --- | --- |
| abfA | -.739** | -.701** | -.481* | .635** | -.554** |
| lig | -0.294 | -.482* | -.647** | .694** | -.778** |
| cex | -.504* | -0.342 | -0.267 | .486* | -.669** |
| pgu | -0.027 | -.508* | -0.363 | .468* | -0.308 |
| cdh | -0.155 | -0.234 | -0.248 | 0.348 | -0.106 |
| glx | -0.154 | -0.409 | -.522* | .645** | -0.19 |
| manB | -0.308 | -.481* | -.554** | .486* | -.543** |
| mnp | -0.272 | -.528* | -.622** | .656** | -.549** |
| sga | -.594** | -.494* | -0.413 | .566** | -.732** |
| chiA | -.696** | -.555** | -0.348 | 0.407 | -.744** |
| gcd | -0.167 | 0.217 | 0.124 | -0.213 | -0.174 |
| xylA | -.563** | -.689** | -.703** | .755** | -.669** |
| apu | -0.034 | -.436* | -0.42 | .467* | -0.048 |
| iso-plu | -.529* | -.678** | -.579** | .607** | -0.312 |
| aclB | -.466* | -.581** | -.660** | .589** | -0.329 |
| mct | -0.343 | -.714** | -.683** | .646** | -0.356 |
| rbcL | -.490* | -0.401 | -0.312 | 0.024 | -.582** |
| frdA | -.717** | -.519* | -.624** | .552** | -.704** |
| korA | -.434* | -.675** | -.491* | .614** | -0.287 |
| accA | -.486* | -0.182 | -0.157 | 0.278 | -.698** |
| acsA | -.437* | -.487* | -0.375 | .544** | -.566** |
| acsE | -0.407 | -.668** | -.712** | .764** | -.562** |
| pccA | -.525* | -.708** | -.719** | .696** | -.671** |
| smtA | -.623** | -.723** | -.660** | .691** | -.733** |
| mmoX | -0.268 | -.582** | -.567** | .518* | -0.252 |
| mxaF | -.613** | -.702** | -.645** | .553** | -.651** |
| pmoA | -0.32 | -.653** | -.753** | .680** | -.563** |
| pqq-mdh | -.746** | -.648** | -.466* | .440* | -.470* |
| amoA1 | -.517* | -.640** | -.536* | 0.254 | -.449* |
| nirS2 | -.575** | -0.289 | -0.12 | -0.064 | -0.121 |
| nosZ1 | -.725** | -.633** | -.522* | .570** | -.494* |
| ureC | -.613** | -0.286 | -.462* | 0.402 | -.628** |
| narG | -.739** | -0.32 | -0.087 | -0.036 | -0.123 |
| nifH | -.694** | -0.375 | -0.299 | 0.275 | -0.387 |
| nirK2 | -.829** | -.435* | -0.173 | 0.375 | -.453* |
| nirS1 | -.551** | -0.215 | -0.003 | -0.158 | -0.024 |
| amoA2 | -0.352 | -.657** | -.443* | .511* | -0.232 |
| napA | -0.318 | -.663** | -.602** | .592** | -.490* |
| nirS3 | -0.346 | -.534* | -.493* | .644** | -.640** |
| nosZ2 | -.812** | -.482* | -0.29 | 0.338 | -0.384 |
| amoB | -.518* | -.700** | -.735** | .816** | -.605** |
| nirK1 | -.712** | -.496* | -0.177 | 0.306 | -.481* |
| nirK3 | -.680** | -.567** | -0.258 | 0.318 | -0.317 |
| gdhA | -.741** | -.469* | -0.294 | 0.263 | -.606** |
| bpp | -0.177 | -0.294 | -0.27 | 0.329 | -0.413 |
| phoX | -0.318 | -0.417 | -.450* | .521* | -.558** |
| pqqC | -.663** | -0.282 | -0.282 | 0.238 | -.472* |
| phnK | -.649** | -.715** | -.602** | .613** | -.549** |
| phoD | -.638** | -.495* | -.643** | .533* | -.769** |
| ppx | -0.331 | -.608** | -.650** | .439* | -.457* |
| apsA | -0.319 | -.567** | -.709** | .744** | -.504* |
| soxY | -.477* | -.482* | -.563** | .529* | -0.365 |
| dsrA | -.763** | -.527* | -0.362 | .497* | -.553** |
| yedZ | -.445* | -0.129 | -0.235 | 0.283 | -.574** |

**Supplementary Table 10.** Major topological properties of phylogenetic molecular ecological networks of functional genes and keystone species in *A*. *palmeri* (AP) and native (N) rhizosphere soils.

| network | AP | N |
| --- | --- | --- |
| node_number | 351 | 486 |
| edge_number | 765 | 1217 |
| net.average.degree | 4.358974 | 5.00823 |
| net.positive | 372 | 339 |
| net.negative | 393 | 878 |
| edge.density | 0.012454 | 0.010326 |
| net.diameter | 10 | 8 |
| net.mean_distance | 4.409735 | 3.91319 |
| Assortativity | -0.3963 | -0.44512 |
| net.degree.centrality | 0.236117 | 0.253591 |
| average_neighbors | 31.34913 | 54.32737 |
| net.modularity | 0.550435 | 0.572774 |
| r2.power.law | 0.839 | 0.779 |

**Supplementary Table 11.** Soil physicochemical properties under invasive *A*. *palmeri* and native vegetation within 22 sampling plots.

| Variables | *A*. *palmeri* plots (AP) | |  | native plots (N) | |  | *p*-value |
| --- | --- | --- | --- | --- | --- | --- | --- |
|  | Means ± standard deviations | Variation coefficients (%) |  | Means ± standard deviations | Variation coefficients (%) |  | AP vs N |
| pH | 8.30±0.18 | 2.24 |  | 8.33±0.24 | 2.85 |  | 0.389 |
| Total N (g·kg^-1^) | 1.4±0.77 | 55.11 |  | 1.65±1.08 | 65.24 |  | 0.197 |
| Total C (g·kg^-1^) | 21.47±9.87 | 45.99 |  | 29.64±22.40 | 75.59 |  | **0.002** |
| Total P (g·kg^-1^) | 0.155±0.08 | 55.34 |  | 0.172±0.07 | 42.66 |  | 0.642 |
| N-NH_4_ (mg·kg^-1^) | 24.76±11.02 | 44.51 |  | 29.18±20.21 | 69.26 |  | **0.041** |
| N-NO_3_ (mg·kg^-1^) | 21.99±14.17 | 64.48 |  | 18.86±14.23 | 75.42 |  | 0.451 |
| P-PO_4_ (mg·kg^-1^) | 24.22±14.02 | 57.91 |  | 25.41±20.46 | 80.50 |  | 0.114 |
| Soil C:N ratio | 16.79±5.30 | 31.58 |  | 19.89±14.54 | 73.08 |  | **0.026** |
| Soil C:P ratio | 167.54±92.33 | 55.11 |  | 184.81±149.41 | 80.85 |  | 0.238 |
| Soil N:P ratio | 10.43±5.81 | 55.74 |  | 10.18 ±5.49 | 53.97 |  | 0.913 |

**Supplementary Table 12.**

| nodeid | net.d  egree | net.  closeness | hubs | authority | modulars | Neighborhood  Connectivity | net.  betweenness |
| --- | --- | --- | --- | --- | --- | --- | --- |
| OTU_437 | 27 | 0.000878735 | 0.440299 | 0.440299 | 1 | 10.33333 | 10648.74 |
| OTU_1155 | 15 | 0.000813008 | 0.38472 | 0.38472 | 1 | 14.53333 | 2201.922 |
| OTU_1461 | 14 | 0.000974659 | 1 | 1 | 3 | 33.64286 | 3980.135 |
| OTU_1690 | 13 | 0.000974659 | 0.992294 | 0.992294 | 3 | 36.07692 | 3197.103 |
| OTU_2132 | 13 | 0.000821018 | 0.518742 | 0.518742 | 3 | 20.46154 | 1582.892 |
| OTU_2148 | 12 | 0.000869565 | 0.25325 | 0.25325 | 1 | 13.33333 | 7220.369 |
| OTU_508 | 10 | 0.000894454 | 0.888092 | 0.888092 | 3 | 40.3 | 1320.767 |
| OTU_38 | 9 | 0.000825083 | 0.528719 | 0.528719 | 3 | 27.33333 | 1883.812 |
| OTU_92 | 9 | 0.000869565 | 0.782071 | 0.782071 | 3 | 38.88889 | 936.8458 |
| OTU_187 | 8 | 0.000838926 | 0.769961 | 0.769961 | 3 | 43.25 | 806.8569 |
| OTU_31 | 8 | 0.000703235 | 0.066502 | 0.066502 | 1 | 7.5 | 197.0926 |
| OTU_419 | 8 | 0.0008726 | 0.798292 | 0.798292 | 3 | 44.5 | 808.1422 |
| OTU_5394 | 8 | 0.000853242 | 0.633684 | 0.633684 | 1 | 37.75 | 1086.019 |
| OTU_661 | 8 | 0.000830565 | 0.639278 | 0.639278 | 3 | 33.75 | 469.8592 |
| OTU_767 | 8 | 0.000791139 | 0.285827 | 0.285827 | 1 | 20.25 | 1100.99 |
| OTU_872 | 8 | 0.000877193 | 0.61273 | 0.61273 | 3 | 37 | 1107.368 |
| OTU_148 | 7 | 0.000775194 | 0.249135 | 0.249135 | 3 | 17.28571 | 1924.137 |
| OTU_1992 | 7 | 0.000679348 | 0.012292 | 0.012292 | 1 | 5.428571 | 2951.498 |
| OTU_222 | 7 | 0.000787402 | 0.261444 | 0.261444 | 1 | 19.57143 | 656.3313 |
| OTU_4 | 7 | 0.000902527 | 0.743806 | 0.743806 | 3 | 48.14286 | 841.2526 |
| OTU_51 | 7 | 0.000892857 | 0.635496 | 0.635496 | 2 | 42.57143 | 784.9908 |
| OTU_1023 | 6 | 0.000854701 | 0.651717 | 0.651717 | 2 | 50.66667 | 600.8045 |
| OTU_10374 | 6 | 0.000775194 | 0.407168 | 0.407168 | 3 | 28.66667 | 181.1881 |
| OTU_1213 | 6 | 0.000834725 | 0.356717 | 0.356717 | 3 | 30.16667 | 706.2182 |
| OTU_1328 | 6 | 0.000854701 | 0.651717 | 0.651717 | 2 | 50.66667 | 600.8045 |
| OTU_1583 | 6 | 0.000696379 | 0.067614 | 0.067614 | 1 | 10.16667 | 173.1646 |
| OTU_1622 | 6 | 0.000822368 | 0.466667 | 0.466667 | 3 | 34 | 344.7884 |
| OTU_1738 | 6 | 0.000726744 | 0.031011 | 0.031011 | 1 | 7.166667 | 214.1449 |
| OTU_3116 | 6 | 0.000701262 | 0.046 | 0.046 | 1 | 7.666667 | 112.8841 |
| OTU_3658 | 6 | 0.000735294 | 0.26821 | 0.26821 | 3 | 23.16667 | 554.3072 |
| OTU_4266 | 6 | 0.000730994 | 0.299842 | 0.299842 | 3 | 23.66667 | 493.219 |
